# A Mechanically Robust In-Situ Solidified Polymer Electrolyte for SiO_x_-Based Anodes Toward High-Energy Lithium Batteries

**DOI:** 10.1007/s40820-025-01759-4

**Published:** 2025-05-08

**Authors:** Cizhen Luo, Huanrui Zhang, Chenghao Sun, Xing Chen, Wenjun Zhang, Pengzhou Mu, Gaojie Xu, Rongxian Wu, Zhaolin Lv, Xinhong Zhou, Guanglei Cui

**Affiliations:** 1 College of Chemistry and Molecular Engineering, Qingdao University of Science and Technology, Qingdao, 266042 People’s Republic of China; 2 Qingdao Institute of Bioenergy and Bioprocess Technology, Qingdao Industrial Energy Storage Research Institute, Chinese Academy of Science, Qingdao, 266101 People’s Republic of China; 3 Shandong Energy Institute, Qingdao, 266101 People’s Republic of China; 4 Qingdao New Energy Shandong Laboratory, Qingdao, 266101 People’s Republic of China

**Keywords:** High-energy lithium batteries, SiO_x_-based anodes, Polymer electrolyte, Micro-phase separation structure, Cycle performance

## Abstract

Silicon suboxide (SiO_x_, 0 < x < 2) is an appealing anode material to replace traditional graphite owing to its much higher theoretical specific capacity enabling higher-energy-density lithium batteries. Nevertheless, the huge volume change and rapid capacity decay of SiO_x_ electrodes during cycling pose huge challenges to their large-scale practical applications. To eliminate this bottleneck, a dragonfly wing microstructure-inspired polymer electrolyte (denoted as PPM-PE) is developed based on in-situ polymerization of bicyclic phosphate ester- and urethane motif-containing monomer and methyl methacrylate in traditional liquid electrolyte. PPM-PE delivers excellent mechanical properties, highly correlated with the formation of a micro-phase separation structure similar with dragonfly wings. By virtue of superior mechanical properties and the in-situ solidified preparation method, PPM-PE can form a 3D polymer network buffer against stress within the electrode particles gap, enabling much suppressed electrode volume expansion and more stabilized solid electrolyte interface along with evidently decreased electrolyte decomposition. Resultantly, PPM-PE shows significant improvements in both cycling and rate performance in button and soft package batteries with SiO_x_-based electrodes, compared with the liquid electrolyte counterpart. Such a dragonfly wing microstructure-inspired design philosophy of in-situ solidified polymer electrolytes helps facilitate the practical implementation of high-energy lithium batteries with SiO_x_-based anodes.

**Supplementary Information:**

The online version contains supplementary material available at 10.1007/s40820-025-01759-4.

## Highlights

- Inspired by the microstructure of high-mechanical-property dragonfly wings, a phase separation structure design philosophy of in-situ solidified polymer electrolytes was first proposed, enabling superior mechanical properties of them.
- By virtue of superior mechanical properties and the in-situ solidified preparation method, the failure behaviors of SiO_x_-based electrodes are effectively suppressed by the as-developed polymer electrolyte.
- A dragonfly wing microstructure-inspired polymer electrolyte (denoted as PPM-PE) enables excellent electrochemical performance of SiO_x_-based electrodes in button-type and soft package full cells.

**Supplementary Information:**

The online version contains supplementary material available at 10.1007/s40820-025-01759-4.

## Graphical abstract

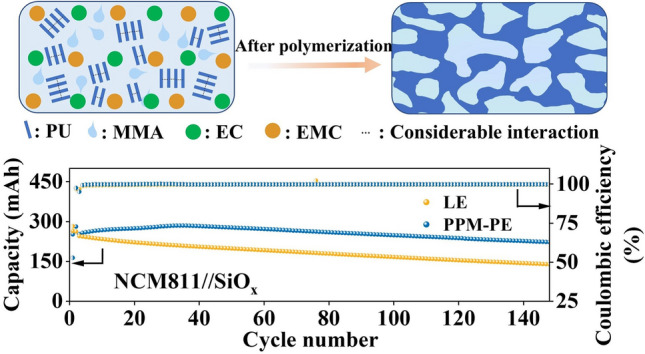

## Introduction

In recent years, lithium-ion batteries (LIBs) technology has driven the industry progress of consumer electronics, electric vehicles, drones and power tools, which in turn, exacerbates the market need for higher-energy–density LIBs [1]. To raise the energy density of LIBs, high-specific-capacity anode materials are being developed as one important strategy [2]. Anode materials such as silicon (Si) and silicon suboxide (SiO_x_) have garnered significant attention as alternatives to conventional graphite for next-generation LIBs, since they offer significantly higher theoretical specific capacities (3800 ~ 4200 mAh g^‒1^ for Si and 2200 ~ 2500 mAh g^‒1^ for SiO_x_ vs. only 372 mAh g^‒1^ for graphite) [3, 4]. In contrast to Si, SiO_x_ exhibits relatively lower volume expansion (~ 200%), which helps mitigate mechanical stress and improve cycling stability [5]. By balancing capacity retention with improved cycle durability, SiO_x_ is positioned as commercially more viable high-capacity anode materials than Si for next-generation energy storage systems [6]. Unfortunately, SiO_x_ anodes still suffer from electrode structure disintegration and unstabilized solid electrolyte interphase (SEI) along with continuous electrolyte decomposition during cycling [7, 8]. And therefore, a much shortened cycle life of SiO_x_ anodes is obtained, which hinders their progress in practical applications [9, 10].

To optimize the performance of SiO_x_ anodes, various strategies such as liquid organic electrolyte engineering [11, 12], the development of polymer electrolytes [13, 14], the use of surface coating [15–17], and binder optimization [18–20] have been adopted. Among them, the development of polymer electrolytes should be one of the most promising solutions, given their advantages in terms of low cost, easy preparation, good electrochemical properties as well as good potential for practical implementation [21, 22]. Although lots of polymer electrolytes have been developed, to the best of our knowledge, rare of them have been proved to achieve excellent cycle performance of SiO_x_ anodes [23]. One of battery failure behaviors is the incompatible SEI along with electrolyte depletion [24]. Such a behavior is highly correlated with the poor mechanical properties of polymer electrolytes, which cannot accommodate the huge volume changes that occur in SiO_x_ anodes during cell cycling [25]. It is worth mentioning that polymer electrolytes, which possess high mechanical properties while achieving superior cycling performance of SiO_x_ anodes, have been never reported in soft package full battery applications [26, 27]. To overcome this barrier, innovative design philosophies of polymer electrolytes are urgently required for SiO_x_ anodes.

**Fig. 1:**
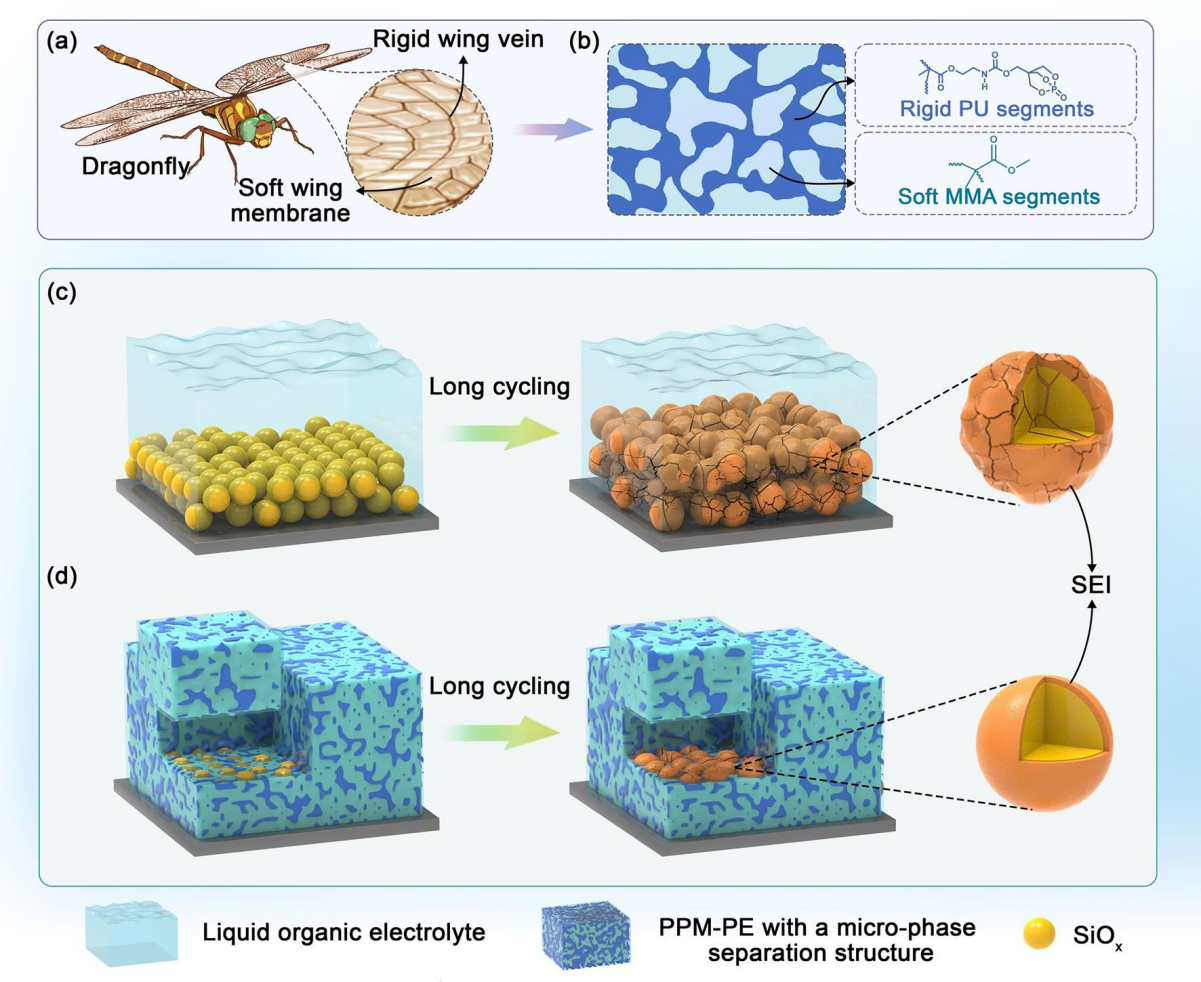
Schematic illustration of **a** dragonfly wings with a phase-separation structure and **b** the as-designed PPM-PE with a phase-separation structure based on soft and rigid polymer segments. Schematic diagram of SiO_x_ electrodes with **c** LE and** d** PPM-PE before and after long cycling

In nature, dragonflies have excellent flight capabilities, highly correlated with dragonfly wings featuring high mechanical properties [28]. In dragonfly wings, there is a phase-separation structure composed of the rigid wing vein phase and soft wing membrane phase (Fig. 1a). The rigid wing vein phases are aligned in specific directions to provide high strength and stiffness, enabling the wing to withstand strong wind resistance and airflow impacts during flight; while the soft wing membrane characterized by rich elastin renders good elasticity and toughness [29], enabling the wing to recover when it encounters external forces and to effectively avoid damage that may be triggered by localized stress concentrations. It has been proved that the unique phase-separated structure endows dragonfly wings with excellent mechanical properties [30, 31].

Inspired by the microstructure of dragonfly wings, we introduce the phase separation mechanism into the design of in-situ solidified polymer electrolytes. Thereupon, a novel polymer electrolyte (denoted as PPM-PE) with excellent mechanical properties was developed via in-situ polymerization of bicyclic phosphate ester- and urethane motif-containing monomer (PU) and methyl methacrylate (MMA) in commercially available liquid organic electrolyte. By precisely modulating the intermolecular interactions between monomers within the electrolyte precursor, PPM-PE can form a micro-phase separation structure (Fig. 1b), evidenced by transmission electron microscopy (TEM) imaging, differential scanning calorimeter (DSC) and small angle X-ray scattering (SAXS) analyses. In the micro-phase separation structure of PPM-PE, PU segments and MMA motifs mimic the rigid wing vein phases and soft wing membrane phase of dragonfly wings, respectively. PPM-PE films exhibit an elongation at break of 161% and a breaking strength of 1.58 MPa, much superior to the electrolyte counterparts based on poly(PU) and poly(MMA) (denoted as PPU-PE and PMMA-PE, respectively). Moreover, PPM-PE also possesses excellent electrochemical properties such as a large lithium transference number (${\text{t}}_{{\text{Li}}^{+}}$) and high oxidation resistance, and a good flame-retardant ability. It is demonstrated that benefitted by its superior mechanical properties and the in-situ solidified preparation method, PPM-PE can effectively suppress the excessive volume expansion of SiO_x_ electrodes and form more stable, compatible SEI, contrasting sharply with the much thickened SiO_x_ electrode and unstabilized SEI when using traditional liquid organic electrolytes (Fig. 1c, d). Resultantly, PPM-PE enables significantly improved electrochemical performance of SiO_x_-based electrodes in button-type and soft package full cells, demonstrating good potential for practical applications.

## Experimental Section

### Materials

Anhydrous dichloromethane and triethylamine were purchased from Aladdin Inc. 2-isocyanatoethyl methacrylate (IM) were purchased from Meryer Chemical Technology Co., Ltd. 4-hydroxymethyl-2,6,7-trioxa-1-phosphabicyclo [2.2.2] octane 1-oxide (HP) was purchased from TCI Co., Ltd. MMA, 2,2-azobisisobutyronitrile (AIBN), hydrochloric acid, sodium chloride, sodium bicarbonate and anhydrous magnesium sulfate were purchased from Aladdin Inc. The electrolyte 1 M LiPF_6_ in ethylene carbonate/diethyl carbonate (EC/DMC) (1:1, v/v) with 10 vol% fluoroethylene carbonate (FEC) was purchased from Duoduo Reagent Co., Ltd.

### Synthesis of PU

Firstly, 100 mL dichloromethane and 5.0 g HP were added to a three-mouth round-bottomed flask in an ice bath with argon gas. After stirring for 5 min, 0.5 mL trimethylamine followed by 4.31 g IM was added into the solution above. This reaction was performed for 12 h. After the reaction, the obtained organic liquid was washed twice with 30 mL 1 wt% hydrochloric acid, twice with deionized water, twice with 30 mL 1 wt% sodium hydroxide solution, twice with deionized water, and then once with aqueous saturated sodium chloride solution. After drying with an appropriate amount of anhydrous magnesium sulfate, the organic liquid was concentrated by using a rotary evaporator to remove excess solvent. Finally, 20 mL of dichloromethane was used to dissolve the crude solid, and 80 mL of n-hexane was added for recrystallization. After filtration and vacuum drying at room temperature, PU monomer was obtained.

### Synthesis of PPM-PE

PPM-PE was prepared via in-situ copolymerization of PU and MMA monomers dissolved in the commercially available liquid electrolyte at certain weight ratios. After the mixture of electrolyte components mentioned above, AIBN with 0.5 wt% of monomer weight was added into this solution. The electrolyte precursor was then treated under 60 °C for 6 h to give PPM-PE.

### Preparation of Electrodes

The SiO_x_ electrode slurry was prepared by mixing aqueous polyacrylic acid(PAA) binder with super P and SiO_x_ at a weight ratio of 1: 1: 8. The well-mixed SiO_x_ electrode slurry was scraped on the copper foil using a doctor blade, followed by drying in a vacuum oven at 100 °C for 24 h. LiNi_0.8_Co_0.1_Mn_0.1_O_2_(NCM811) cathodes were prepared by mixing NCM811, super P and PVDF binder with a mass ratio of 8:1:1. Then the slurry was blade-coated on an aluminum foil current collector and dried at 60 °C for 24 h under vacuum. The electrodes were cut into required size before used.

### Cell Assembly and Electrochemical Measurements

The coin-type battery was assembled in a glove box with strict water and oxygen control (both H_2_O and O_2_ contents < 0.1 ppm). The fabrication of in-situ generated coin-type (CR2032) Li//SiO_x_ half batteries were prepared as follows: SiO_x_ electrodes were cut into circular disks with 14 mm in diameter with a mass loading of 1.13 mg cm^−2^ and Celgard 2500 porous polypropylene (PP) membrane as the separator. The soft package cell was prepared by a stacking method using NCM811 cathode (7.0 mg cm^−2^) and SiO_x_ anode (1.13 mg cm^−2^) with PP membrane as the separator. Finally, these batteries were kept at 60 °C for 6 h to complete the polymerization. Linear scan voltammetry (LSV) tests and electrochemical impedance spectroscopy (EIS) measurements were conducted on a multichannel BioLogic VMP-300 workstation in a coin cell. Cyclic voltammetry (CV) measurements were performed between 0.001 and 1.5 V with scan rates of 0.2, 0.4, 0.6, 0.8, and 1 mV s^−1^.

### Other Characterization Techniques

The structures of polymers were characterized by nuclear magnetic resonance (NMR) spectroscopy and Fourier transform infrared (FT-IR) spectrometers. The prepared electrode samples were analyzed morphologically using a scanning electron microscope (Hitachi Model S-4800) and a cross-sectional ion mill-scanning electron microscope (IM-SEM, Hitachi IM4000PLUS). The hardness and modulus of the electrode were analyzed by nanoindentation test. A universal testing machine (MTS, E43) was used to analyze the mechanical properties of the polymer electrolyte film. Atomic force microscopy (AFM), TEM, X-ray photoelectron spectroscopy (XPS) and time-of-flight secondary ion mass spectrometry (TOF–SIMS) were used to characterize the surface morphology or surface chemical composition of the electrodes.

### Interaction Energy Calculation

The interaction energy ${E}_{\text{int}}$ represents the strength of the interaction between the components in the system and is obtained from the following equation:

1 $${E}_{\text{int}}={E}_{\text{total}}-\sum {E}_{\text{component}}$$

$$E_{{{\text{total}}}} :{\text{ Total }}\,{\text{energy}}\,{\text{of}}\,{\text{the}}\,{\text{system}};\,\,\,\,E_{{{\text{component}}}} :{\text{ the}}\,{\text{energy}}\,{\text{of}}\,{\text{each}}\,{\text{component}}\,{\text{in}}\,{\text{the}}\,{\text{system}}.$$

### Finite Element Simulation

This program uses an electrochemical model solution in COMSOL based on Newman’s theory of porous electrodes.

2 $${j}_{n}={j}_{0 }\left\{\text{exp}\left(\frac{{\alpha }_{\text{a}}F}{RT}\eta \right)-\text{exp}\left(-\frac{{\alpha }_{\text{c}}F}{RT}\eta \right)\right\}$$

3 $$\eta ={\varnothing }_{\text{S}}-{\varnothing }_{\text{l}}-{E}_{\text{eq}}$$

where: ${j}_{0}$ is the exchange current density; *η* is the local overpotential; *α*_c_ and *α*_a_ are the electrochemical reaction transfer coefficients of the anode and the cathode, which are taken as 0.5; F is the Faraday’s constant; R is the ideal gas constant. *ϕ*_s_ is the solid-phase potential;* ϕ*_l_ is the liquid-phase potential; and E_eq_ is the equilibrium potential of the material.

The exchange current density expression is:

4 $${j}_{0}=F{{{k}_{0}c}_{\text{l}}^{{\alpha }_{\text{a}}}\left({c}_{\text{s},\text{max}}-{c}_{\text{s},\text{sur}f}\right)}^{{\alpha }_{\text{a}}}{c}_{\text{s},\text{surf}}^{{\alpha }_{\text{c}}}$$

where: k_0_ is the reaction rate constant; *c*_s,max_ is the maximum solid-phase lithium ion concentration of the material; *c*_s,surf_ is the lithium ion concentration at the interface between the electrode and the electrolyte.

Solid-phase Ohm’s law and liquid-phase Ohm’s law, respectively:

5 $${i}_{\text{l}}=-{\delta }_{\text{l}}^{\text{eff}}\nabla {\varnothing }_{\text{l}}+\frac{2{\delta }_{\text{l}}^{\text{eff}}RT}{F}\left(1+\frac{\partial \text{ln}f}{{\text{ln}c}_{\text{l}}}\right)\left(1-{t}_{+}\right)\nabla \left({\text{lnc}}_{\text{l}}\right)$$

6 $${i}_{\text{s}}=-{\delta }_{\text{s}}^{\text{eff}}\nabla {\varnothing }_{\text{s}}$$

where: *i*_s_ and *i*_l_ represent solid-phase electron current and liquid-phase ion current, respectively; ${\updelta }_{\text{s}}^{\text{eff}}$ and ${\updelta }_{\text{l}}^{\text{eff}}$ represent solid-phase and liquid-phase potentials, respectively; and t_+_ is the migration number.

The mass conservation equation for lithium ions in the solid phase is described by Fick’s second law as:

7 $$\frac{\partial {c}_{\text{s}}}{\partial \text{t}}=\frac{{D}_{\text{s}}}{{r}^{2}}\bullet \frac{\partial }{\partial r}\left({r}^{2}\bullet \frac{\partial {c}_{\text{s}}}{\partial r}\right)$$

where: *c*_s_ represents the solid-phase lithium ion concentration; *D*_s_ is the solid-phase diffusion coefficient of lithium ions; *r* is the radius of the spherical particles.

The mass conservation equation for lithium ions in the liquid phase is described by the concentrated solution theory as:

8 $$\upepsilon \frac{\partial {c}_{l}}{\partial t}=\nabla \bullet \left({D}_{\text{l}}^{\text{eff}}\nabla {c}_{\text{l}}\right)+\frac{1-{t}_{+}}{F}\bullet {J}_{n}$$

9 $${D}_{\text{l}}^{\text{eff}}={D}_{\text{l}}\bullet {\varepsilon }^{\text{brugg}}$$

where: ${\varepsilon }_{\text{l}}$ is the liquid phase volume fraction in the electrode and separator; ${D}_{\text{l}}^{\text{eff}}$ is the equivalent diffusion coefficient in the electrolyte, corrected by the Bragman coefficient.

## Results and Discussion

### Design, Synthesis and Characterization of PPM-PE

To mechanically stabilize the structure of SiO_x_ anodes, the in-situ solidified preparation of polymer electrolytes should be a better option since polymer monomers can enter into the electrode particles gap and form a 3D polymer network buffer against stress after polymerization [32]. The construction of phase-separated structure has also been investigated in many polymer materials [33, 34], which can effectively improve the mechanical modulus and/or stretchability of polymer materials. However, how to construct in-situ solidified polymer electrolytes with a phase separation structure similar with dragonfly wings has been never developed for SiO_x_ anodes. To obtain them, it is crucial to design and prepare thermodynamically metastable-state dual polymer phase within traditional liquid organic electrolyte. For this purpose, theoretically it is feasible to design copolymers of two monomers that show quite different interaction strengths [35]. Meanwhile, the design of polymer monomers needs to meet the requirements of good electrochemical properties (e.g., wide electrochemical stability window, high ionic conductivity and large ${\text{t}}_{{\text{Li}}^{+}}$) of polymer electrolytes. Therefore, PPM-PE based on in-situ polymerization of PU and MMA monomers was designed based on following considerations: (1) The free radical polymerization reaction based on acrylate monomers was chosen owing to the high polymerization conversion [35, 36]. (2) MMA with a medium dielectric constant was chosen since PMMA-based polymer electrolytes show high ionic conductivities and relatively wide electrochemical stability windows. (3) PU monomer was designed in consideration of that the bicyclic phosphate ester with a large dielectric constant can form strong hydrogen bonding with urethane motifs; this results in the larger interaction strength between PU monomers than those between PU and MMA, as well as between MMA monomers, enabling PU self-aggregation.

**Fig. 2:**
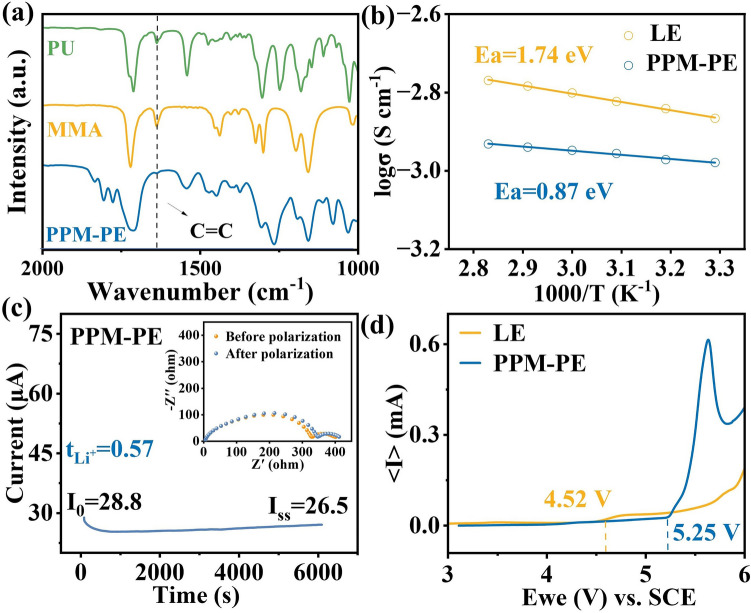
**a** Partial amplified FT-IR spectra of PU, MMA and PPM-PE. **b** Liner fitting plot for σ of LE and PPM-PE ranging from 30 to 80 °C. **c** Chronoamperometry curve during polarization of Li|PPM-PE|Li symmetrical batteries. Inset: EIS of Li|PPM-PE|Li symmetrical cell. **d** LSV curves of PPM-PE and LE

PU monomer was synthesized via a simple nucleophilic addition reaction of HP and IM catalyzed by triethylamine (Fig. S1). The chemical structure of PU monomer was confirmed through ^1^H NMR and FT-IR spectroscopy analyses **(**Figs. S2 and S3). As shown in Fig. S4, the preparation of PPM-PE involves the in-situ copolymerization of PU and MMA monomers within the electrolyte 1 M LiPF_6_ in EC/DMC (1:1 in vol) with 10 vol% FEC (denoted as LE). FT-IR spectroscopy analyses show that the characteristic peak of typical C = C at about 1625 cm^–1^ cannot be observed in the solidified electrolyte, confirming the successful preparation of PPM-PE (Figs. 2a and S5).

To optimize the electrolyte component ratios, polymer electrolytes with varied PU:MMA molar ratios (9:1, 7:3, 5:5) and varied LE to polymer matrix weight ratios (5:1, 6:1, 7:1, 8:1) were constructed. By preliminary performance comparisons in Li//SiO_x_ button cells (Fig. S6) and EIS tests (Fig. S7a-c), PPM-PE with a PU: MMA molar ratio of 9:1 and a LE-to-polymer matrix weight ratio of 7:1 was selected as the model for subsequent studies.

Ionic conductivity is a vital parameter measuring electrolyte performance. Figure S8 shows the ionic conductivity of varied electrolytes at different temperatures. Notably, the room-temperature ionic conductivity of PPM-PE is 1.03 × 10^–3^ S cm^–1^, basically meeting the practical applications requirements. The ionic transport mechanism of these electrolytes can be described by the Arrhenius equation [37]:

10 $$\sigma \left(T\right)={\sigma }_{0}\text{exp}\left[-{E}_{\text{a}}/RT\right]$$

where σ is the ionic conductivity, ${\upsigma }_{0}$ represents a pre-exponential factor, R is the gas constant, *E*_a_ indicates the activation energy, and *T* is absolute temperature. As determined from Eq. (10), the *E*_a_ value of PPM-PE is 0.87 eV, which is lower than that (1.74 eV) of LE (Fig. 2b). This finding indicates that PPM-PE has a lower Li^+^ migration barrier. Meanwhile, the ${\text{t}}_{{\text{Li}}^{+}}$ of PPM-PE achieves 0.57 (Fig. 2c), contributing to the reduction of concentration polarization and the enhancement of rate performance [38]. Using stainless steel as the working electrode and a lithium metal electrode as the counter electrode, LSV was tested. As observed in Fig. 2d, PPM-PE has a higher oxidation decomposition potential of exceeding 5.25 V, much higher than that (4.52 V) of LE. Moreover, PPM-PE also shows a good flame-retardant ability (Fig. S9).

**Fig. 3:**
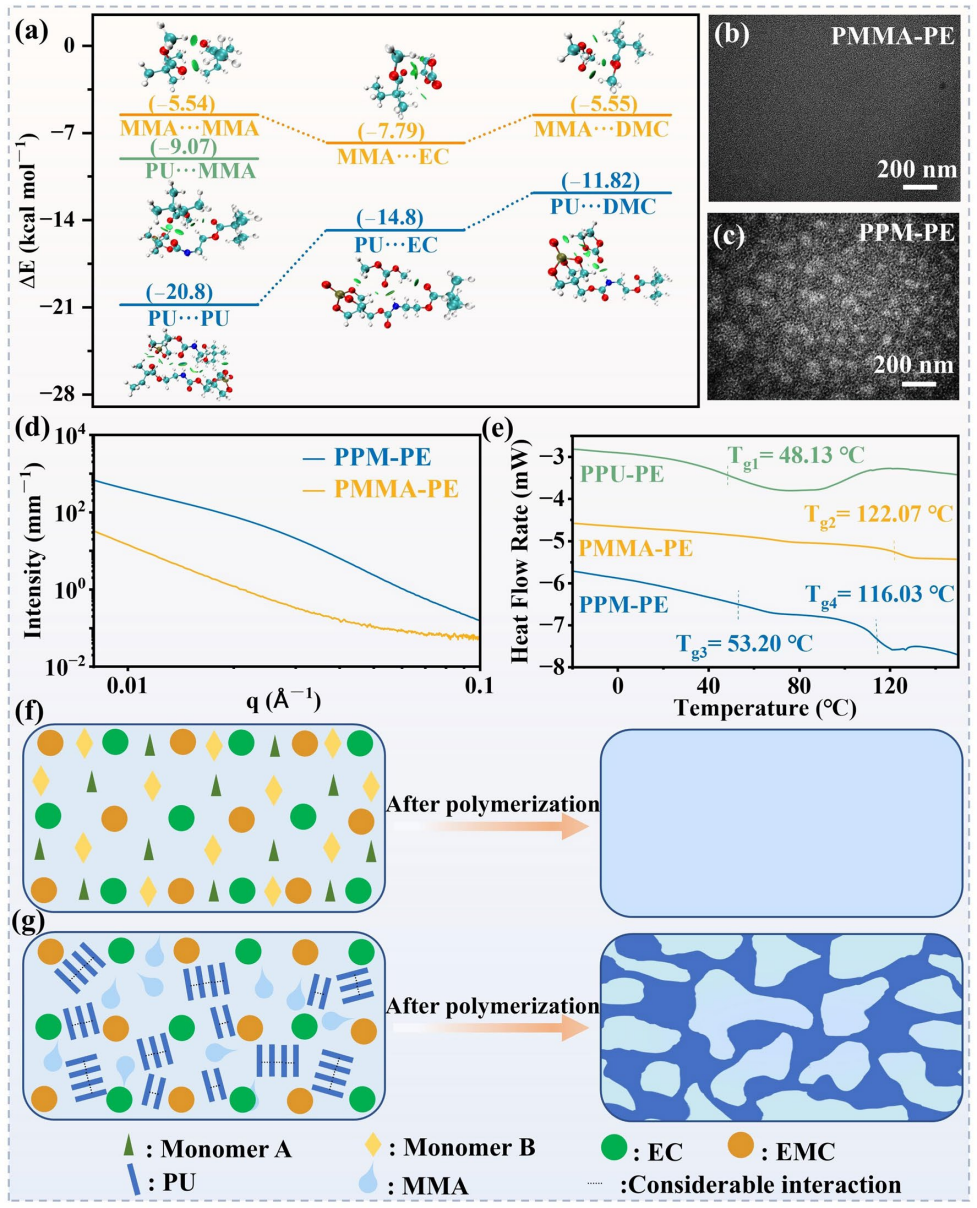
**a** Calculations of intermolecular binding energy in the PPM-PE precursor performed at the B3LYP-D3(BJ)/def2-SVP level of theory (Green plates show noncovalent interactions at the isovalue surface of ${\updelta }_{\text{g}}^{\text{inter}}$ 0.005 in IGMH analysis). TEM images of **b** PMMA-PE film, **c** PPM-PE film. **d** SAXS curves of PMMA-PE and PPM-PE films. **e** DSC curves of PPM-PE, PPU-PE and PMMA-PE. Schematic illustrations of condensed structure formation of **f** traditional in-situ solidified polymer electrolyte and **g** PPM-PE

As mentioned above, one key factor for the formation of phase separation structures of in-situ formed polymer electrolytes lies in how to precisely modulate the intermolecular interactions so that allows monomer motifs self-aggregation within liquid organic electrolytes [39]. Theoretically, the relatively large difference in molecular polarity and binding energy between the monomers help to form monomer motifs self-aggregation, and subsequently to construct a phase separation structure of polymer electrolytes [40, 41]. To prove the design rationality of PPM-PE, the dielectric constant (ε′) of monomers was measured via a capacitance method at different frequencies. As can be seen from Fig. S10, the ε′ of PU and MMA differ evidently throughout the frequency range, indicating a considerable difference in polarity between the two monomers. Moreover, the intermolecular binding energy involving the two polymer monomers in the PPM-PE precursor was investigated via first principles analyses. As shown in Fig. 3a, the binding energy value between PU monomers (PU···PU) was − 20.8 kcal mol^–1^, much lower than that between MMA monomers (MMA···MMA, − 5.54 kcal mol^–1^) and between PU and MMA (PU···MMA, − 9.07 kcal mol^–1^). Noting that the binding energy value of PU···PU is also smaller than those between PU with any carbonate solvent in PPM-PE. The considerable differences in monomer ε′, and interaction strengths between monomers as well as between monomers with carbonates in the PPM-PE precursor allow monomer motifs self-aggregation and thus help to form a phase separation structure.

To verify whether PPM-PE forms a phase separation structure, TEM imaging was used. As can be observed in Fig. 3b, PMMA-PE films exhibit a homogeneous surface morphology. In contrast, a nanoscale micro-phase separation morphology occurs in PPM-PE films after PU monomer was introduced (Fig. 3c). Such a finding is further supported via SAXS analyses (Fig. 3d). It is evident that PMMA-PE films show no significant scattering peaks, demonstrating that no phase separation occurs. Whereas, PPM-PE copolymer exhibits a broad scattering peak around 0.01–0.04 Å^–1^, indicative of the formation of a phase separation structure, in line with our design philosophy. Moreover, as shown in Fig. 3e, the glass transition temperatures (*T*_g_) of PMMA-PE and PPU-PE measured by DSC are 122.07 and 48.13 °C, respectively. Since two *T*_g_ values can be observed at 53.2 and 116.03 °C for PPM-PE, which approximately correspond to the *T*_g_ of PPU-PE and PMMA-PE, respectively. This finding further indicates the formation of a phase separation structure in PPM-PE. Additionally, the small *T*_g_ shifts observed in PPM-PE compared with corresponding PMMA-PE and PPU-PE means that MMA motifs are slightly miscible with PU segments in PPM-PE. These results fully confirm the formation of a micro-phase separation morphology of PPM-PE, indicative of our design rationality.

Figure 3f, g vividly presents the condensed structure formation difference of traditional in-situ solidified polymer electrolyte and PPM-PE. In the precursor of traditional in-situ solidified polymer electrolyte, monomers do not show evident difference in interaction strengths between them (i.e., A···A, B···B and A···B), and therefore monomer self-aggregation does not occur (Fig. 3f). Therefore, this precursor will form a polymer electrolyte with a homogeneous phase structure after polymerization. In comparison, the considerable interaction strength of PU···PU in the precursor of PPM-PE results in PU self-aggregation (Fig. 3g). Subsequently, a phase separation structure generates after polymerization in PPM-PE.

### Mechanical Properties Characterization of Electrolytes

**Fig. 4:**
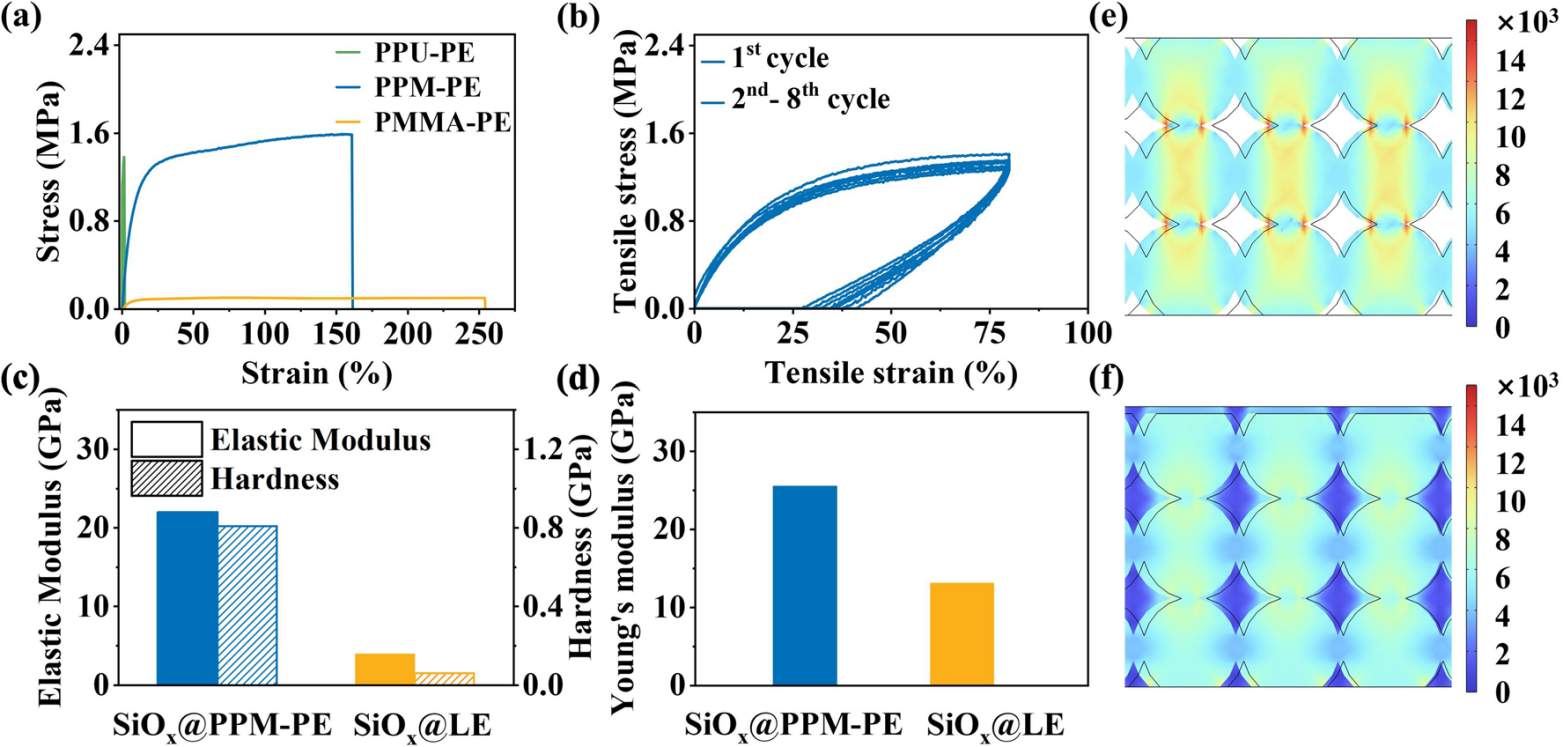
**a** Stress–strain curves of varied electrolyte films. **b** Stress–strain curves for cyclic tensile tests of PPM-PE films under 80% elongation. **c** Surface elastic modulus and hardness of SiO_x_ electrodes disassembled from half-cells using varied electrolytes after 100 cycles obtained via nanoindentation test. **d** Surface Young’s modulus of SiO_x_ electrodes disassembled from half-cells using different electrolytes after 100 cycles measured by AFM. Stress distribution of SiO_x_ electrodes in the presence of **e** LE and **f** PPM-PE obtained via finite elements simulation

The mechanical properties of varied electrolytes were characterized through the stress–strain curves (Fig. 4a). It can be seen that PMMA-PE films display excellent tensile properties with an elongation at break of more than 254%, while its breaking strength is only 0.10 MPa. The low breaking strength is attributed to the highly solvated polymer chains and insufficient interchain interactions of PMMA within LE. While PPU-PE films presents a high Young’s strength (1.4 MPa) but an ultralow breaking elongation (negligible). In sharp contrast, PPM-PE films show excellent mechanical properties with an elongation at break of 161% and a breaking strength of 1.58 MPa. This is mainly benefited from a micro-phase separation structure composed of the soft MMA motif phase and rigid PU motif phase, which mimic the wing membrane phase and the wing vein phases of dragonfly wings, respectively. It is noteworthy that the stress corresponding to PPM-PE films rapidly increases with increased strain and then changes gently until broken off, belonging to a mechanical behavior of hard and tough materials. This mechanical behavior helps to withstand the huge volume expansion and stabilize the SEI of SiO_x_ electrodes during cycling. To further test the mechanical responsiveness of PPM-PE, we used cyclic loading–unloading measurements with a strain limit of 80% and set the interval between each cycle to 1 h. The stress values remain approximately constant over the 8 cycles, indicating that PPM-PE has a favorable recovery capability (Fig. 4b). It is well-known that dissipated energy reflects the ability of a material to resist damage under cyclic loading. At 80% tensile strain, the initial cycle dissipation energy of PPM-PE films is 0.68 MJ m^–3^ with a loss coefficient of 77% (Fig. S12). While at the 8th cycle, the dissipation energy remains at 0.55 MJ m^–3^ with a loss coefficient of about 72% (Fig. S12). Less decrease in dissipation energy upon cyclic loading–unloading indicates the superior fatigue resistance of PPM-PE.

As mentioned above, in-situ formed polymer electrolytes can enter into the electrode particles gap and form a 3D network buffer against stress after polymerization. Additionally, the polymer matrix of them can participate in forming a polymer-reinforced mechanically robust SEI layer. These behaviors help to promote the electrode cohesion and mechanically stabilize the electrode structure and interface [42]. To prove the improved electrode cohesion, nanoindentation tests were performed on SiO_x_ electrodes disassembled from half-cells using varied electrolytes. As indicated by the load–displacement curves (Figs. 4c and S13) under a given nanoindentation force, the indentation depth (145 nm) of SiO_x_ electrodes with PPM-PE (denoted as SiO_x_@PPM-PE) is smaller than that (555 nm) of SiO_x_ electrodes with LE (denoted as SiO_x_@LE). Moreover, compared with SiO_x_@LE, much higher elastic modulus (21.9 vs. 3.9 GPa) and hardness (0.8 vs. 0.06 GPa) of SiO_x_@PPM-PE can be observed. This result demonstrates that PPM-PE evidently increases the mechanical strength of SiO_x_ electrodes. This finding can be further supported by AFM measurements using the peak force quantitative nanomechanics (QNM) model. As shown in Figs. 4d and S15, the SiO_x_@PPM-PE surface exhibits a much higher elastic modulus (25.4 GPa) than that (13.0 GPa) of SiO_x_@LE. Higher intrinsic mechanical strength of SiO_x_ electrodes means that PPM-PE can more effectively mitigate the excessive volume expansion of SiO_x_ electrodes allowing them to have the smaller volume deformation and construct more stabilized SEI during repeated cycling.

In order to comprehend the effect of varied electrolytes upon electrode evolution during cycling, the stresses on SiO_x_ electrodes were simulated with finite elements. As shown in Figs. 4e, f and S16, the stress distortion is concentrated at the particle contact site, and the SiO_x_ electrode particle stress in the presence of PPM-PE is much lower than that using LE. These results indicate that PPM-PE can better buffer the stress generated from electrode expansion, thus effectively maintaining the structural integrity of SiO_x_ electrodes.

### Interfacial Evolution Analysis of SiO_x_ Electrodes

**Fig. 5:**
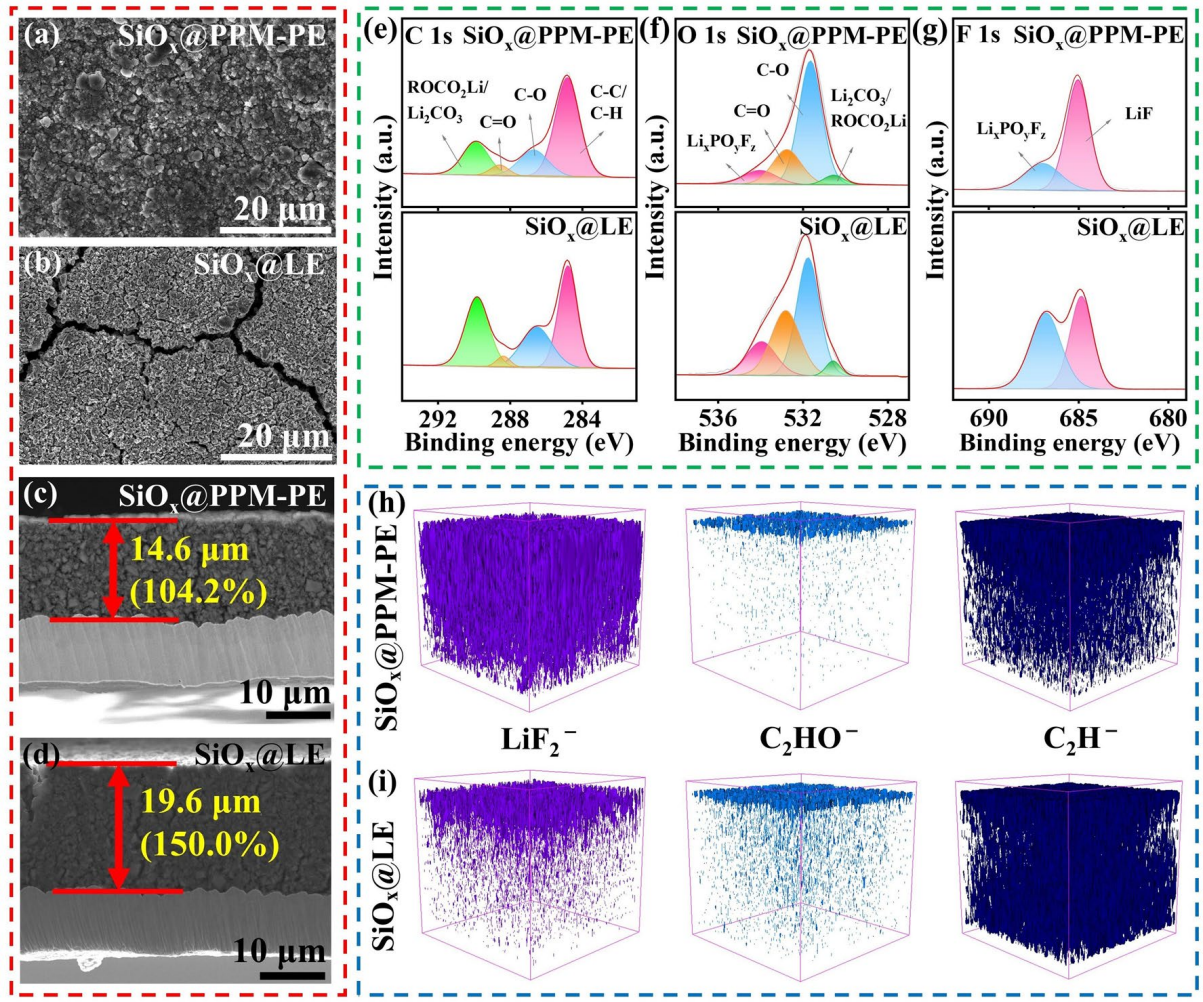
Top-viewed SEM images of **a** SiO_x_@PPM-PE and **b** SiO_x_@LE after 50 cycles at 0.5C rate. Cross-sectional SEM images of **c** SiO_x_@PPM-PE and **d** SiO_x_@LE after 50 cycles at 0.5C rate. XPS spectra in **e** C 1*s*, **f** O 1*s* **g** F 1*s* branches of SiO_x_ electrodes after 30 cycles at 0.5C rate. TOF–SIMS 3D rendering modeling and depth profiling of **h** SiO_x_@PPM-PE and **i** SiO_x_@LE after 50 cycles

To examine the impact of improved mechanical properties of PPM-PE on the SEI stability, we examined the structural evolution of SiO_x_ electrodes with different electrolytes after 50 cycles at 0.5C rate. As shown in Figs. 5a, b and S17a, b, the SiO_x_@PPM-PE surface morphology shows no apparent cracks before and after 50 cycles, while there are large cracks on the surface of SiO_x_@LE after 50 cycles. In addition, scanning electron microscope (SEM) imaging shows that the cross-sectional thickness enhancement of SiO_x_@PPM-PE is only 104.2% (from 14.0 to 14.6 μm) after 50 cycles, much lower than that (150.0%, from 13.0 to 19.6 μm) of SiO_x_@LE (Figs. 5c, d and S18). These observations indicate that PPM-PE significantly inhibits the excessive volume expansion and maintains the structural integrity of SiO_x_ electrodes during cycling.

Suppressed electrode volume expansion is expected to render improved SEI stability in the presence of PPM-PE. To confirm this, TEM imaging coupled with elemental mapping was used to present the morphology of the SEI film formed on the surface of SiO_x_ electrodes after 50 cycles. As shown in Fig. S19, SiO_x_ particles from SiO_x_@PPM-PE form a thin and relatively homogeneous SEI layer on their surface with a thickness of around 18.28 nm, in contrast to the much thicker and more uneven SEI layer with a thickness ranging from 30.54 to 48.07 nm of SiO_x_@LE. It is conceivable that owing to the huge volume change of SiO_x_ electrodes during repeated cycling, SEI layers formed by LE will be frequently damaged, which leads to a huge increase in thickness and inhomogeneity after cycling. With the help of PPM-PE possessing excellent mechanical properties, more stabilized SEI layers characterized by thinner and more even morphologies can be obtained. Additionally, we performed XPS analysis of SEI components on the SiO_x_ electrode surface after 30 cycles. According to the C 1*s* and the O 1*s* spectra (Fig. 5e, f), the Li_2_CO_3_/ROCO_2_Li peak intensity ratio corresponding to electrolyte carbonate decomposition products for SiO_x_@PPM-PE is smaller than that of SiO_x_@LE. Additionally, in the F 1*s* and O 1*s* spectra (Fig. 5f, g), SiO_x_@PPM-PE shows a smaller peak intensity ratio of Li_x_PO_y_F_z_ than SiO_x_@LE, which is derived from electrolyte decomposition. These results confirm that less electrolyte decomposition occurs in the presence of PPM-PE, meaning the formation of more stabilized, compatible SEI. Noting that there is a higher peak intensity ratio of LiF at 685.4 eV in the F 1*s* spectra (Fig. 5g), which is favorable to form a mechanically stable SEI layer [43, 44]. In the N 1*s* spectrum, the characteristic peak of C-N/N–H (399.9 eV) from the polymer matrix of PPM-PE was observed (Fig. S20), meaning that PPM-PE can participate in building SEI layers. The polymer-reinforced SEI layer generally exhibits good mechanical properties, which can, in part, explain the improved mechanical properties of SiO_x_@PPM-PE mentioned above [45]. TOF–SIMS provides more evidences for more stabilized SEI formation in the presence of PPM-PE. Figures 5h, i and S21 present 3D rendered models and depth profiles of specimen fragments during the sputtering process of SiO_x_ electrodes with different electrolytes after 50 cycles at 0.5C rate, where LiF_2_^–^ secondary ions represent LiF and C_2_HO^–^/C_2_H^–^ secondary ions originate from electrolyte carbonate decomposition products. Compared to LE, PPM-PE exhibits higher LiF_2_^–^ content as well as less C_2_HO^–^/C_2_H^–^ content over the entire etching range. These results indicate that PPM-PE can induce the formation of polymer-reinforced SEI layers rich in LiF, which contributes to suppress SiO_x_ electrode volume expansion and electrolyte decomposition during cycling.

### Electrochemical Performance Evaluations of SiO_x_ Electrodes

**Fig. 6:**
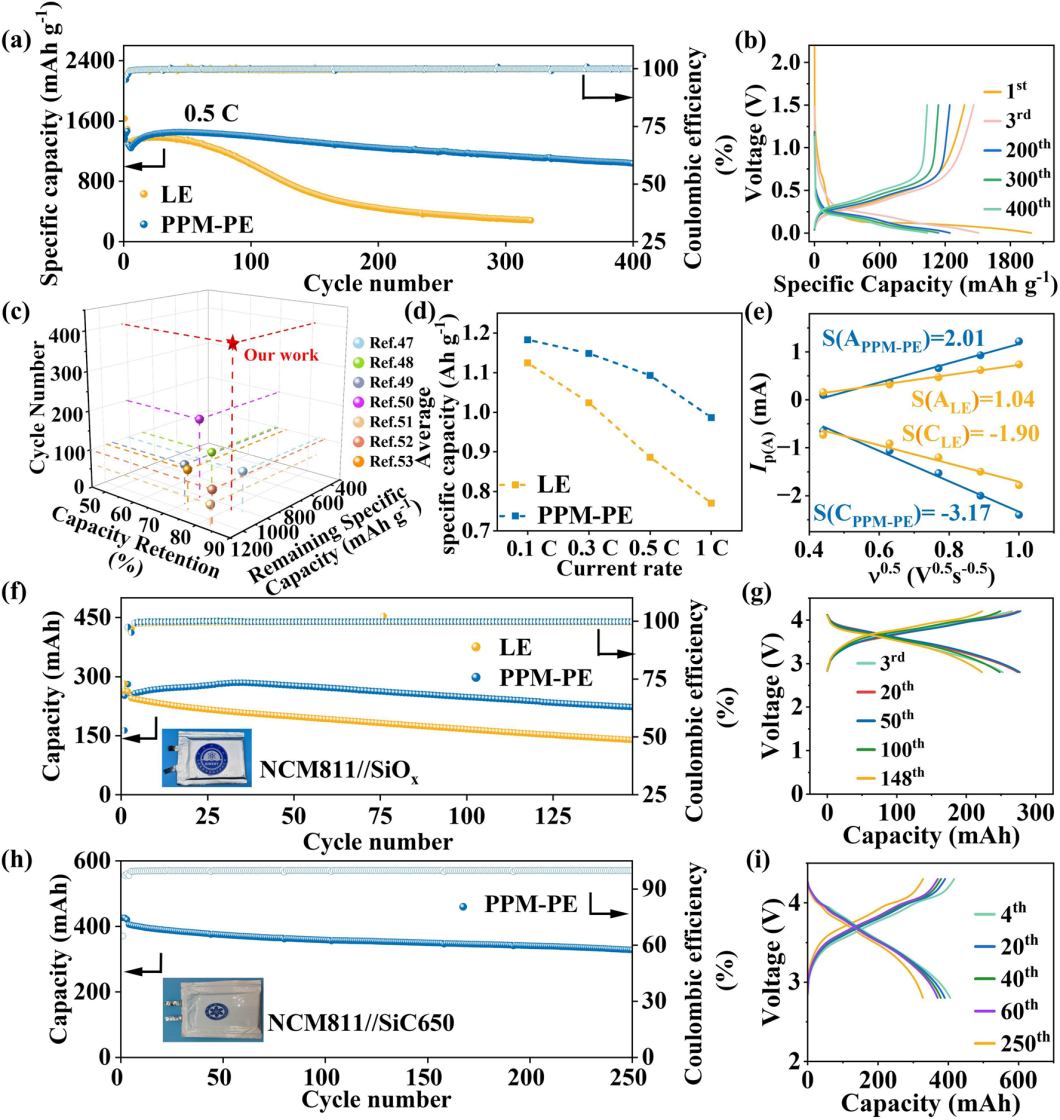
**a** Long-term cycling performance of SiO_x_ (1.13 mg cm^–2^)//Li half-cells with different electrolytes at 0.5C rate under 0.001–1.5 V. **b** Charge–discharge curves of SiO_x_/PPM-PE/Li half-cells at 0.5C rate for varied cycles. **c** Cycle performance comparison of PPM-PE with previously reported typical electrolytes in SiO_x_ electrode-based half-cells. **d** Average specific capacities of SiO_x_ electrode-based half-cells with different electrolytes at different rates. **e** The linear relationships and the slopes between the cathodic and anodic peak current and the square root of the scanning rate for different electrolytes derived from the CV curves. **f** Cycling performance of the NCM811//SiO_x_ soft package full cell with different electrolytes at 1C rate between 2.8 and 4.2 V. The inset is a picture of the as-assembled cell with PPM-PE. **g** Charge and discharge curves of the NCM811/PPM-PE/SiO_x_ soft package full cell at 1C rate for varied cycles. **h** Cycling performance of NCM811//SiC650 soft package full cells at 0.4C rate between 2.8 and 4.3 V and the corresponding **i** charge–discharge curves at varied cycles

To explore the potential application in lithium-ion batteries, the electrochemical performance of the as-developed electrolyte was evaluated in SiO_x_ electrode-based half-cells and soft package full batteries. As shown in Figs. 6a, b and S22, a delithiation capacity of 1035 mAh g^–1^ after 400 cycles corresponding to a capacity retention of 80.63% along with an average Coulombic efficiency of 99.73% can be obtained in the SiO_x_ electrode-based half-cell assembled with PPM-PE at 0.5C rate. Such results are much superior to those of the LE counterpart (capacity retention of 21.26% and average Coulombic efficiency of 99.52%, 320 cycles). The much improved cycling performance is primarily attributed to the more stabilized SEI, in accordance with above-mentioned XPS and TOF–SIMS results. Noting that PPM-PE exhibits superior cycling performance to previously reported typical electrolytes in Li//SiO_x_ button half-cells (Fig. 6c and Table S1) [46–52], definitely confirming the design rationality of PPM-PE.

In addition, PPM-PE also exhibits excellent rate capabilities in SiO_x_ electrode-based half-cells. As shown in Figs. 6d and S23, PPM-PE delivers higher delithiation capacities than LE at different current densities from 0.1C to 1C. For instance, at 1C rate, PPM-PE renders a delithiation capacity of 981.7 mAh g^–1^, apparently exceeding 801.9 mAh g^–1^ of LE. These data demonstrate that PPM-PE enables faster electrochemical reaction kinetics of SiO_x_ electrodes. To prove this, CV of SiO_x_-based half-cells with different electrolytes were performed at different scan rates (Fig. S24). The Li^+^ diffusion coefficient (${\text{D}}_{{\text{Li}}^{+}}$) of SiO_x_ electrodes using different electrolytes can be calculated according to the classical Randels-Sevcik equation [53]:

11 $${I}_{\text{p}}=2.69\times {10}^{5}{n}^{1.5}A{D}_{{\text{Li}}^{+}}^{0.5}{v}^{0.5}{C}_{{\text{Li}}^{+}}$$

where *I*_p_ represents the peak current, A refers to the area of the electrode, ${C}_{{\text{Li}}^{+}}$ expresses the molar concentration of Li^+^, n is the number of charge transfer, and *ν* is the scanning rate. A linear fit of the slope of the cathode/anode peak currents and the square root of the scan rate was utilized to calculate ${\text{D}}_{{\text{Li}}^{+}}$. Clearly, the higher absolute value of slope indicates a higher Li^+^ diffusion coefficient. It is evident that PPM-PE shows a higher absolute value of slope for cathodic and anodic reaction of SiO_x_ electrodes than those of LE (Fig. 6e, −3.17 and 2.01 for PPM-PE vs. −1.90 and 1.04 for LE, respectively). The faster Li^+^ diffusion coefficient indicates faster electrochemical reaction kinetics of SiO_x_ electrodes in the presence of PPM-PE, in line with its superior rate performance.

On the basis, we further explored the feasibility of PPM-PE in practical soft package full cells applications. As shown in Figs. 6f, g and S25, the assembled NCM811 (7.0 mg cm^–2^)//SiO_x_ (1.13 mg cm^–2^) soft-packed cells using PPM-PE maintains 88.14% capacity retention and 99.48% average Coulombic efficiency after 148 cycles at 1C rate, significantly outperforming those of the LE counterpart (56.59% capacity retention and 99.35% average Coulombic efficiency). Additionally, we assembled a 400 mAh NCM811//SiC650 (graphite/SiO_x_ composite electrode with a specific capacity of 650 mAh g^–1^ at 0.1C rate) soft package cell with a commercial-level NCM811 loading of 15 mg cm^–2^. As shown in Fig. 6h, i, the assembled cell provides a capacity retention of 80.81% and an average Coulombic efficiency of 99.68% after 250 cycles at 0.4C rate. These results adequately demonstrate the feasibility of PPM-PE toward practical implementation of SiO_x_-based electrodes. To the best of our knowledge, this is the first case of polymer electrolytes so far to enable excellent cycle performance of SiO_x_ electrodes in soft package full cells.

## Conclusion

In summary, inspired by the dragonfly wing microstructure, we have proposed a phase separation structure design philosophy of in-situ solidified polymer electrolytes to address the serious challenges facing SiO_x_ electrodes toward practical implementation. The as-developed electrolyte was constructed based on the in-situ polymerization of PU and MMA monomers in LE. PMMA-PE possesses excellent electrochemical properties such as a large ${\text{t}}_{{\text{Li}}^{+}}$ (0.57) and a high oxidation decomposition (> 5.25 V), and a good flame-retardant ability. We demonstrated that by precisely modulating the intermolecular interactions between the monomers within the electrolyte precursor, PPM-PE can form a micro-phase separation structure, evidenced by TEM imaging, DSC and SAXS analyses. In the micro-phase separation structure of PPM-PE, PU motifs and MMA segments mimic the rigid wing vein phases and soft wing membrane phase of dragonfly wings, respectively. It is demonstrated that PPM-PE films exhibit an elongation at break of 161% and a breaking strength of 1.58 MPa, much superior to PPU-PE and PMMA-PE. By virtue of superior mechanical properties and the in-situ solidified preparation method, PPM-PE can form a 3D polymer network buffer against stress within the electrode particles gap. Additionally, PPM-PE can form a polymer-reinforced SEI layers rich in LiF. Benefited from these strengths, PPM-PE can effectively suppress the excess volume expansion of SiO_x_ electrodes and construct more stable SEI along with much decreased electrolyte decomposition, compared with traditional LE. Resultantly, PPM-PE enables much improved electrochemical performance of SiO_x_-based electrodes in button-type and soft package full cells. Impressively, the NCM811/PPM-PE/SiO_x_ soft-packed full cell provides 88.14% capacity retention after 148 cycles at 1C rate, and the NCM811/PPM-PE/SiC650 soft package full cell achieves 80.81% capacity retention after 250 cycles at 0.4C rate, demonstrating good potential of PPM-PE for practical applications. Noting that this is the first case of polymer electrolytes so far to enable excellent cycle performance of SiO_x_ electrodes in soft package full cells. This phase separation structure design philosophy of polymer electrolytes explores a new avenue for improving the electrochemical performance and boosting the practical implementation of high-energy LIBs with SiO_x_-based anodes.

## Supplementary Information

Below is the link to the electronic supplementary material.

Supplementary file1 (DOCX 10936 KB)
