# Supplementary material for: A Mechanically Robust In-Situ Solidified Polymer Electrolyte for SiOx-Based Anodes Toward High-Energy Lithium Batteries
Source: Nanomicro Lett. 2025 May 8;17:250. doi: 10.1007/s40820-025-01759-4 (PMC12061835; doi:10.1007/s40820-025-01759-4)
Supplement: Supplementary file 1 — Supplementary file1 (DOCX 10936 KB) [file 40820_2025_1759_MOESM1_ESM.docx]

Supporting Information for

**A Mechanically Robust In-Situ Solidified Polymer Electrolyte for SiO_x_-Based Anodes** **Toward High-Energy Lithium Batteries**

Cizhen Luo^1, 2, 3, 4^, Huanrui Zhang^2, 3, 4^*, Chenghao Sun^2, 3, 4^, Xing Chen^1, 2, 3, 4^, Wenjun Zhang^1, 2, 3, 4^, Pengzhou Mu ^2, 3,4^, Gaojie Xu ^2, 3, 4^, Rongxian Wu^2, 3, 4^, Zhaolin Lv^2, 3, 4^, Xinhong Zhou^1^*, and Guanglei Cui^2, 3, 4^*

^1^ College of Chemistry and Molecular Engineering, Qingdao University of Science and Technology, Qingdao 266042, P. R. China

^2^ Qingdao Industrial Energy Storage Research Institute, Qingdao Institute of Bioenergy and Bioprocess Technology, Chinese Academy of Science, Qingdao 266101, P. R. China

^3^ Shandong Energy Institute, Qingdao 266101, P. R. China

^4^ Qingdao New Energy Shandong Laboratory, Qingdao 266101, P. R. China

*Corresponding authors. E-mail: [zhanghr@qibebt.ac.cn](mailto:zhanghr@qibebt.ac.cn) (Huanrui Zhang); [zhouxinhong@qust.edu.cn](mailto:zhouxinhong@qust.edu.cn) (Xinhong Zhou); [cuigl@qibebt.ac.cn](mailto:cuigl@qibebt.ac.cn) (Guanglei Cui)

**Supplementary Figures**


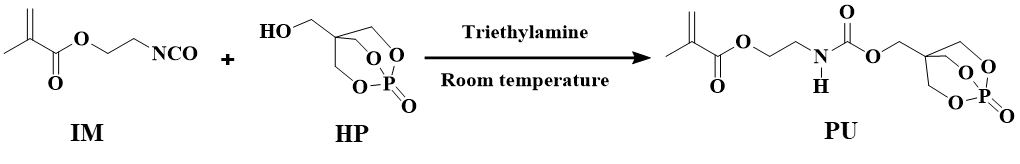


**Fig. S1** Synthesis schematic diagram of PU


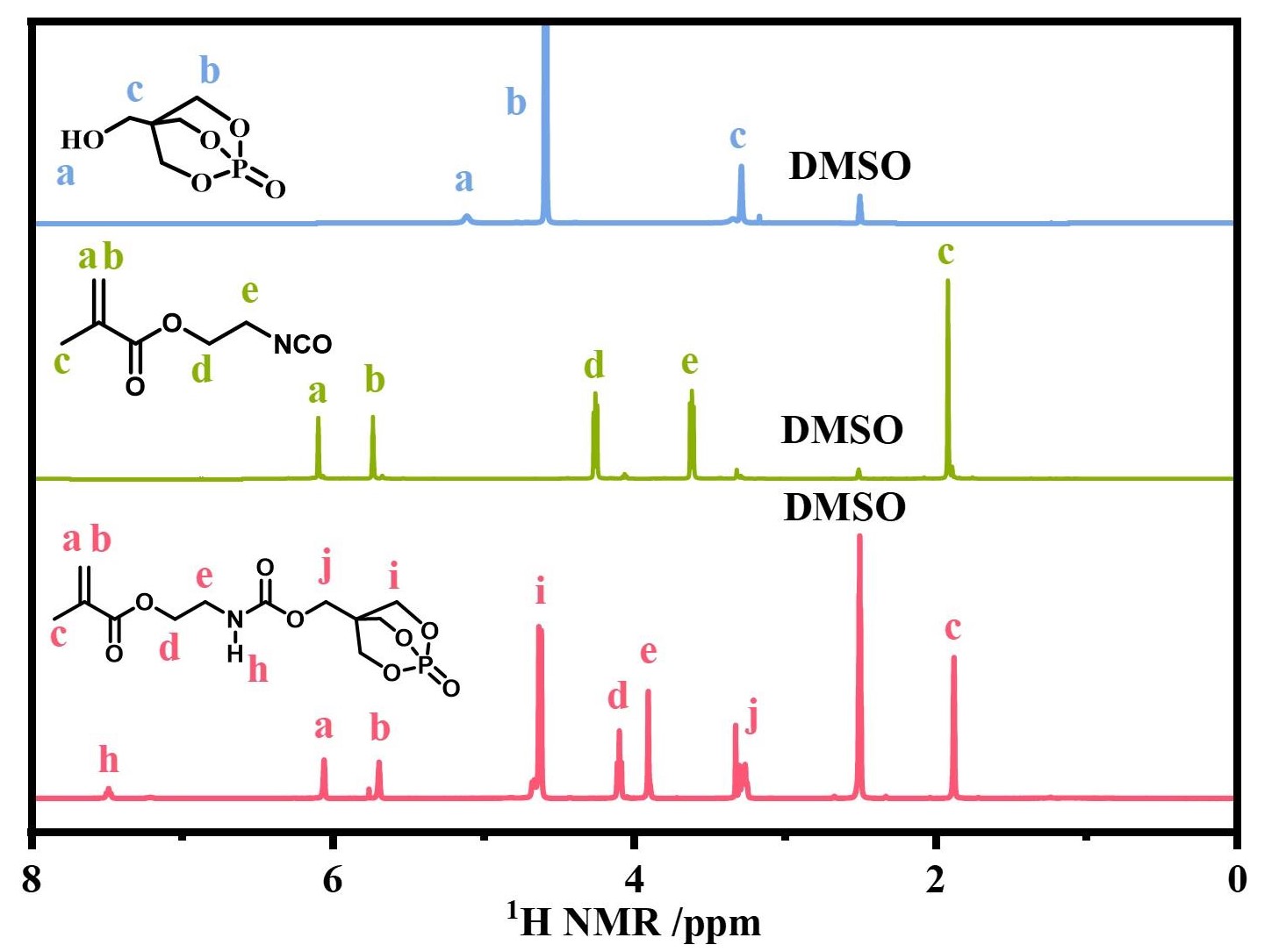


**Fig. S2** ^1^H NMR spectrum of PU and its synthesis reagents

As shown in **Fig. S2**, in the ^1^H NMR spectrum of PU, the chemical shift of hydroxyl proton in HP disappears along with the evident chemical upshift or downshift of H_e_ and H_d_ in IM after reaction, indicative of the successful synthesis of PU.


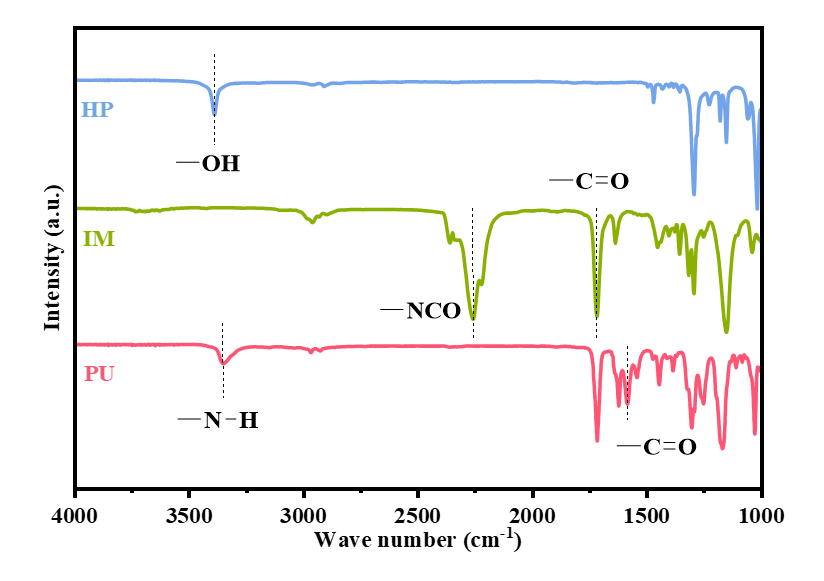


**Fig. S3** FTIR spectra of HP, IM and PU

The disappearance of the characteristic peaks of –NCO at 2274 cm^−1^ and –OH at 3389 cm^−1^ in the FT-IR spectra of IM and HP, and the appearance of the characteristic peak of –C=O and N–H of urethane motifs functional group separately at 1581 and 3308 cm^−1^ prove the successful synthesis of PU monomer.


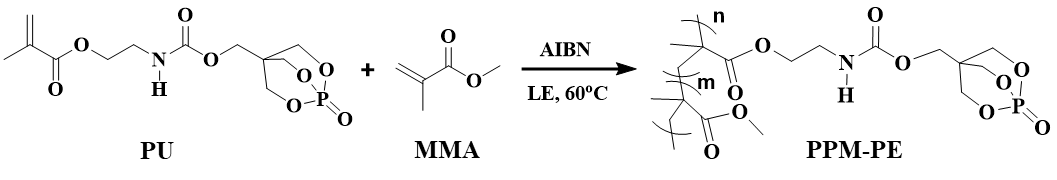


**Fig. S4** Synthesis schematic diagram of PPM-PE


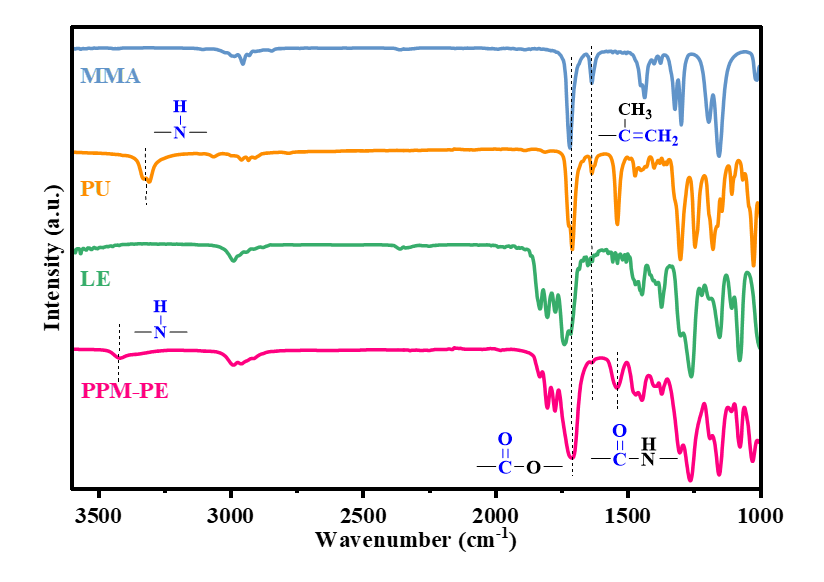


**Fig. S5** FTIR spectra of MMA, PU, LE and PPM-PE

As shown in **Fig. S5**, FTIR spectra show that the typical C=C characteristic peak at about 1625 cm^−1^ was not observed in the prepared polymer electrolyte, which indicates the successful preparation of PPM-PE.


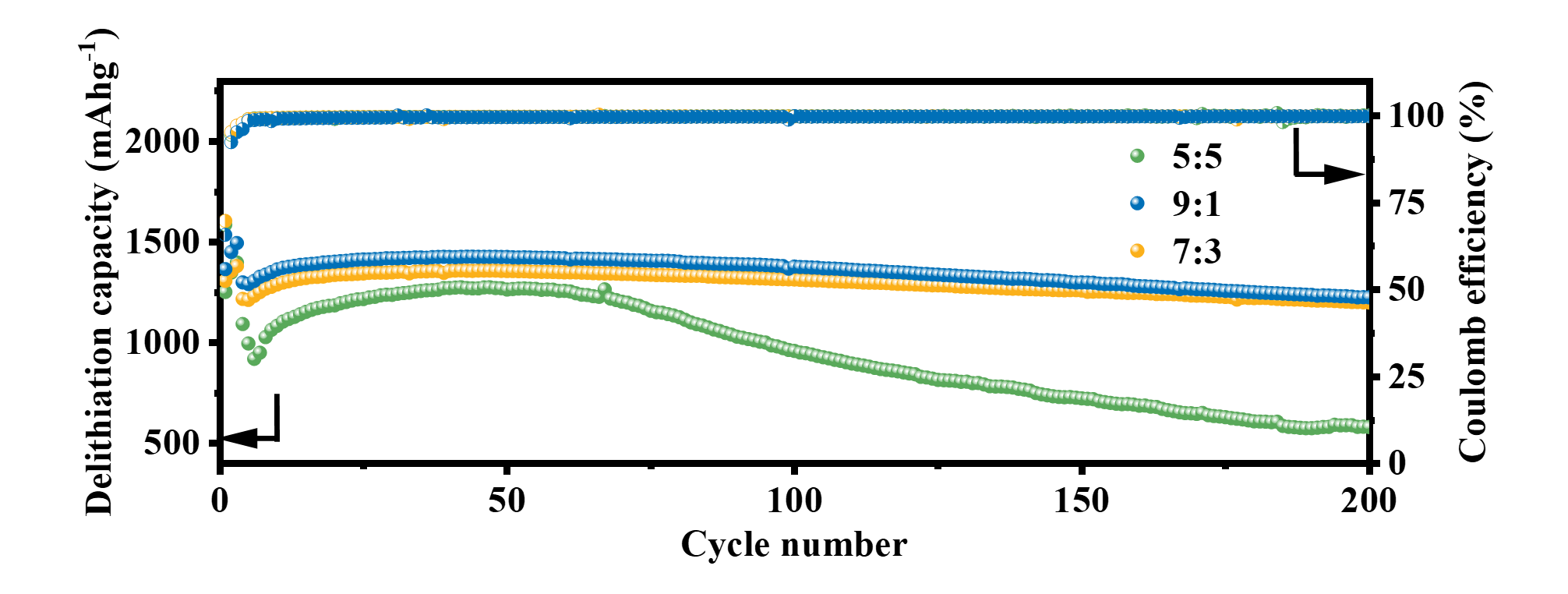


**Fig. S6** Cyclic performance of Li//SiO_x_ button cells with different copolymer monomer molar ratios at 0.5 C rate after 2 cycles of activation at 0.1 C rate and a voltage range of 0.001–1.5 V

The as-designed polymer electrolytes with PU: MMA molar ratios of 9:1, 7:3, and 5:5 is recorded as PE1, PE2, and PE3. As shown in **Fig. S6**, the cell of PE1 has superior cycling performance after 200 cycles compared to PE3 or PE2. Based on these results, a PU: MMA molar ratio of 9:1 (i.e., PE1) was selected as the model for the following research.


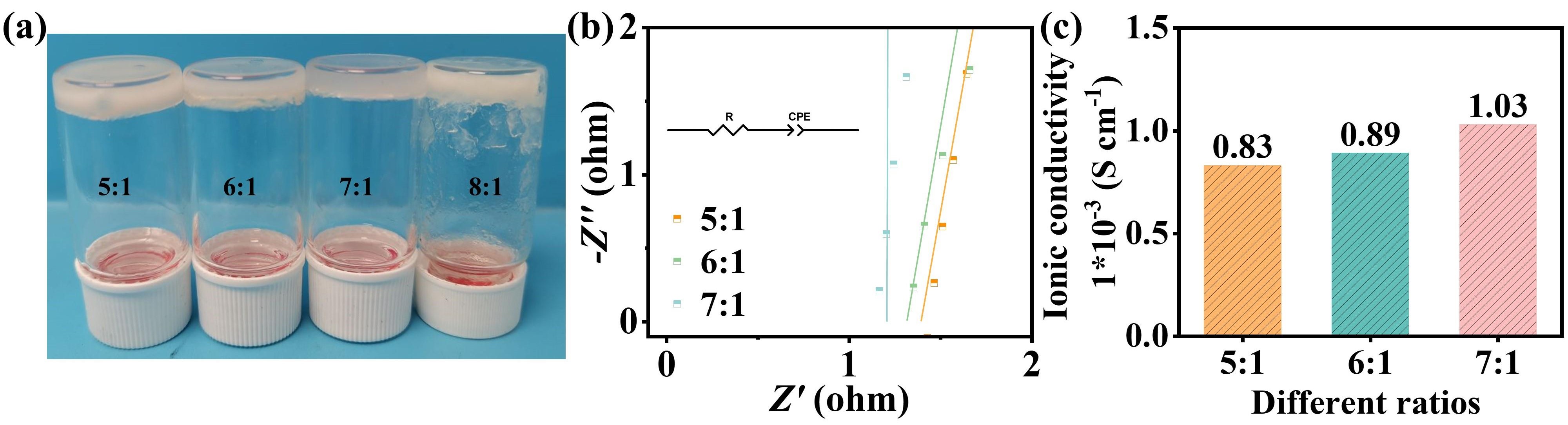


**Fig. S7** **a**) Digital photographic images of polymer electrolytes with different LE to polymer matrix weight ratios, and **b**) electrochemical impedance spectracopy (EIS) of polymer electrolytes with different LE to polymer matrix weight ratios (Inset is an equivalent circuit fitted by Zview software). The dots and lines represent experimental data and fitted data, respectively. **c**) Ionic conductivity of corresponding polymer electrolytes

As shown in **Fig. S7a**, noting that after thermal treatment at 60 °C for 6 h, polymer electrolytes with a weight ratio of LE to polymer matrix of 8:1 are flowable, while the ones with weight ratio of LE to polymer matrix of 7:1, 6:1 and 5:1 exhibit a solidified state.

EIS measurements were performed in the frequency range of 7 MHz to 100 mHz to evaluate the ionic conductivity of polymer electrolytes with varied weight ratios of LE to polymer matrix. PP separators used in these batteries exhibit a measured thickness of 24 μm and an effective surface area of 2.0 cm². As shown in **Fig. S7b**, the weight ratio of LE to polymer matrix of 7:1 demonstrates an optimal ionic conductivity, which was thus set as the optimal electrolyte uptake for following study.


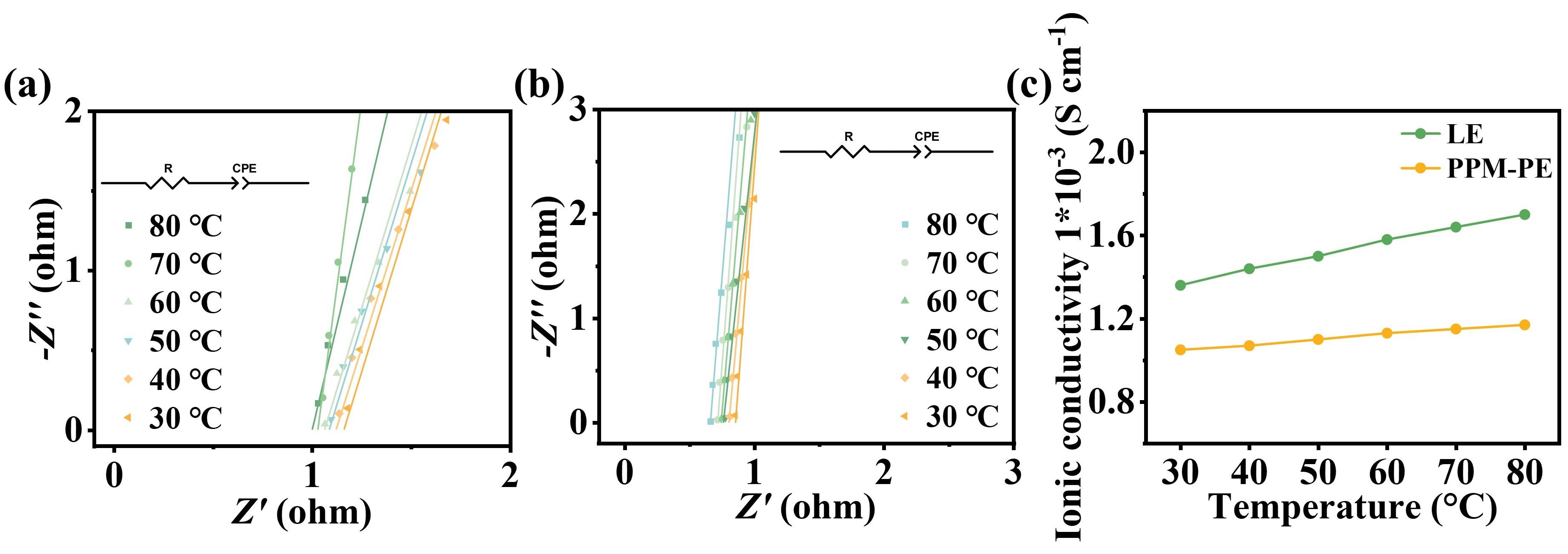


**Fig. S8** EIS measurements **a**) PPM-PE and **b**) LE were performed in the frequency range from 7 MHz to 100 mHz, where the PP separators were measured at a thickness of 24 μm and an effective surface area of 2.0 cm² (Inset is an equivalent circuit fitted by Zview software). The dots and lines represent experimental data and fitted data, respectively. **c**) Temperature-dependent ionic conductivities for LE and PPM-PE

**
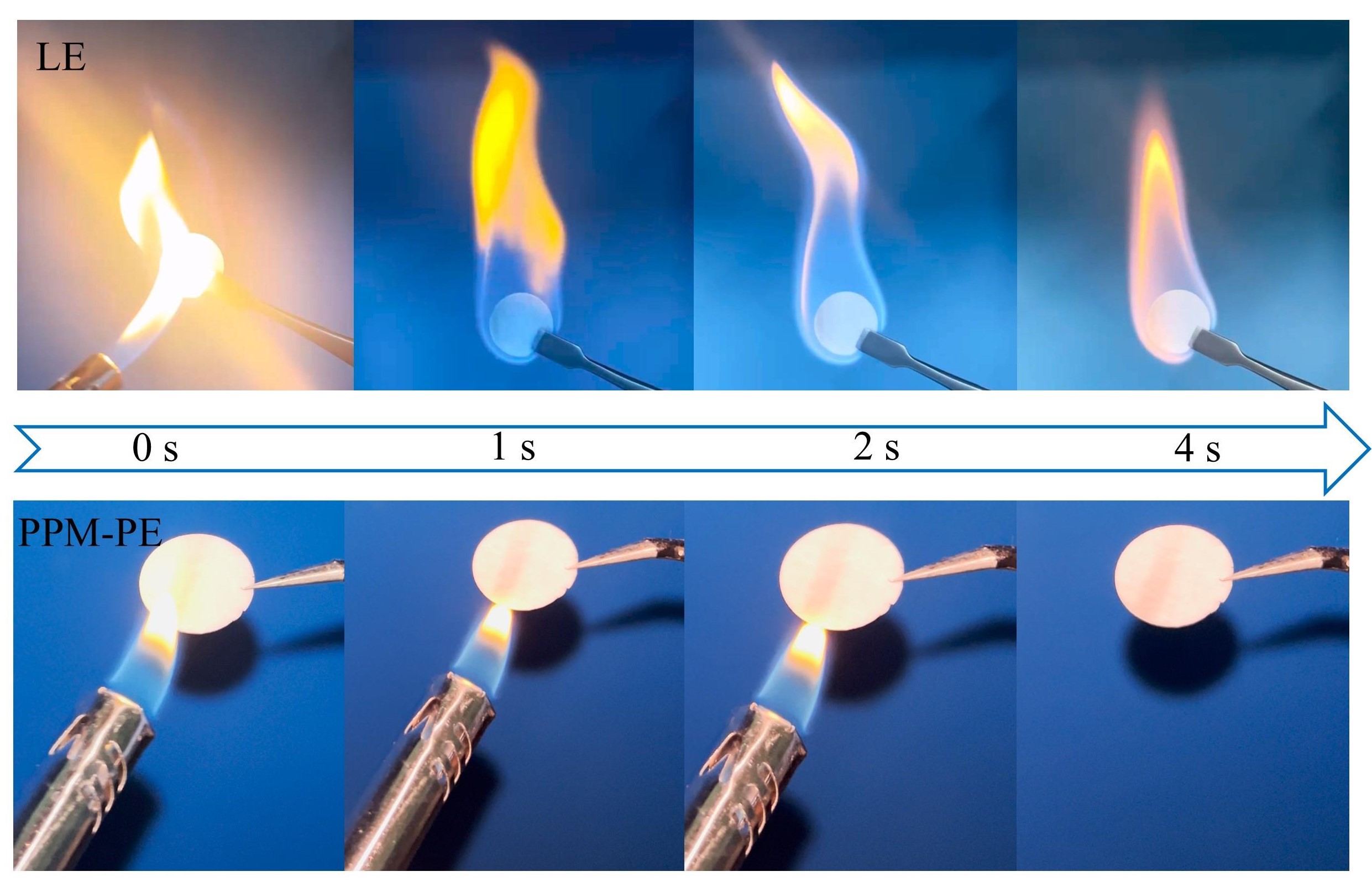
**

**Fig. S9** Flame retardancy test chart for LE and PPM-PE

As illustrated in **Fig. S9**, the separator absorbing LE was ignited rapidly, whereas PPM-PE remained non-ignitable within 3 s. This demonstrates the flame-retardant properties of PPM-PE.


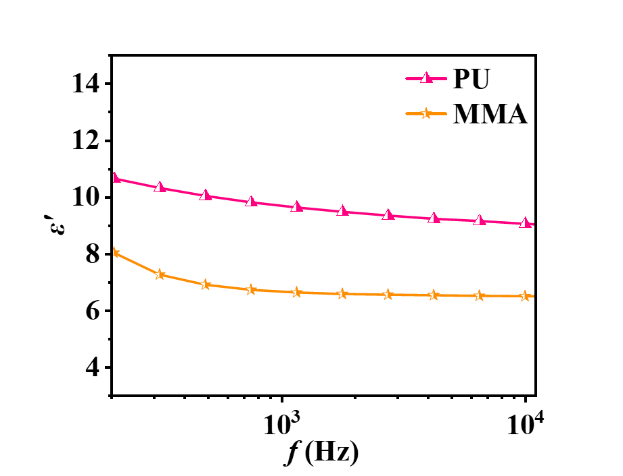


**Fig. S10** Variation of dielectric constant (ε‘) with frequency (f) for MMA and PE

As illustrated in **Fig. S10**, in the entire frequency range, PU and MMA have a large difference in ε′, indicating a large difference in polarity.


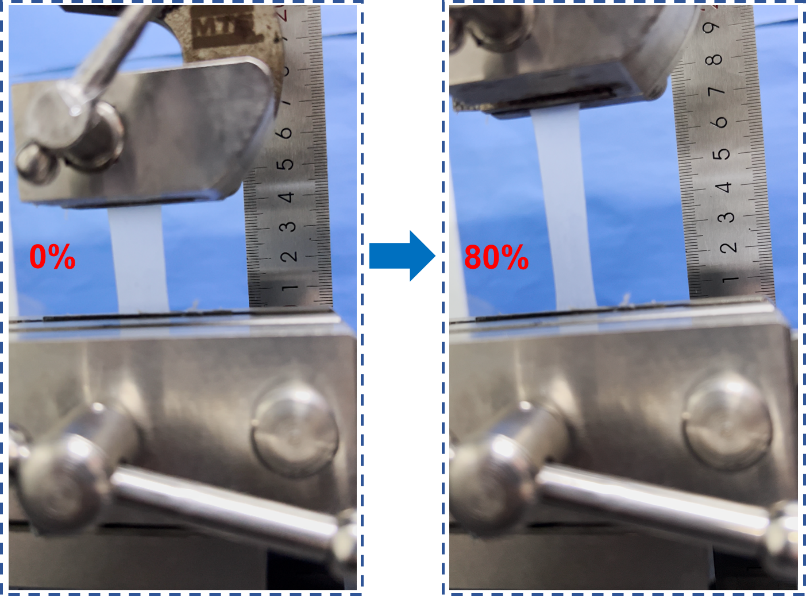


**Fig. S11** Digital images showing the stretching process for PPM-PE films

PPM-PE films show excellent mechanical properties after stretching to 80% of the pristine length.


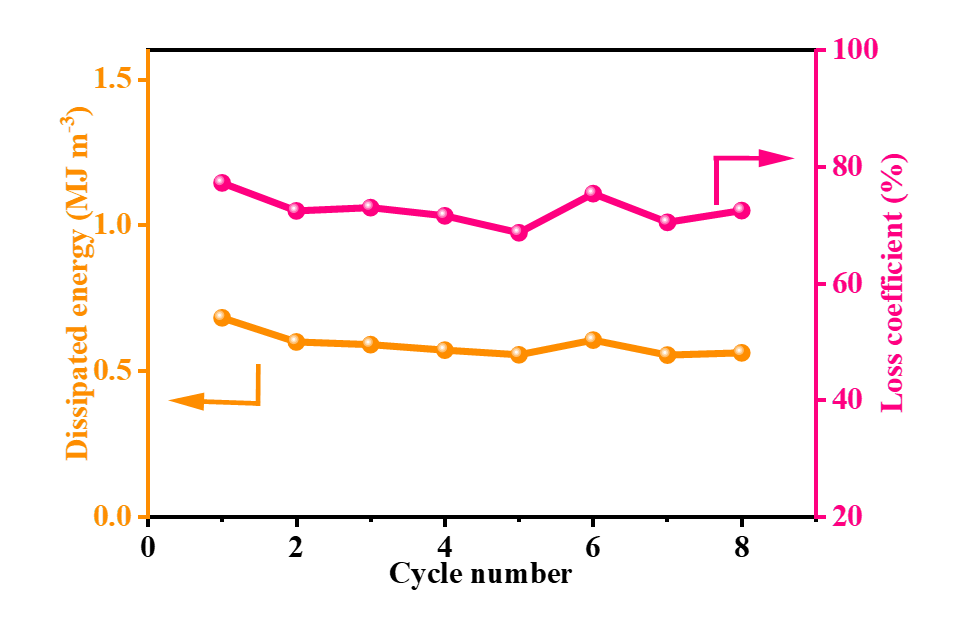


**Fig. S12** The corresponding energy dissipation and loss coefficient of PPM-PE films at per cycle

As shown in **Fig. S12**, at 80% tensile strain, the initial cycle dissipation energy of PPM-PE films is 0.68 MJ m^–^³ with a loss coefficient of 77%. While at the 8^th^ cycle, the dissipation energy remains at 0.55 MJ m^–^³ with a loss coefficient of about 72%.


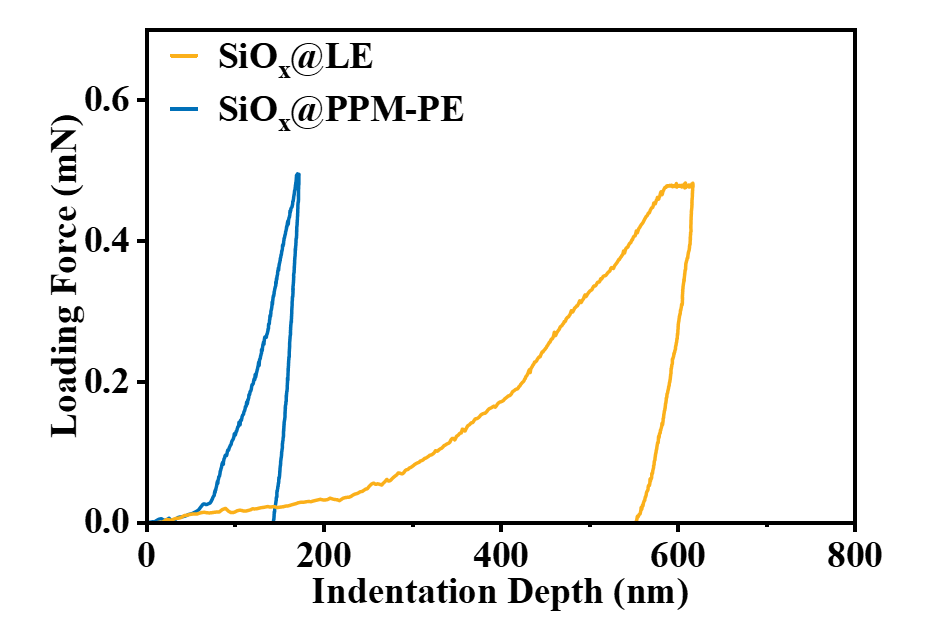


**Fig. S13** Nanoindentation curves of SiO_x_ electrodes disassembled from half-cells with different electrolytes after 100 cycles

As illustrated in **Fig. S13**, under a given nanoindentation force, the indentation depth (145 nm) of SiO_x_@PPM -PE is smaller than that (555 nm) of SiO_x_@LE. This result demonstrates that PPM-PE endows SiO_x_ electrodes with the improved mechanical strength and cohesion than LE.


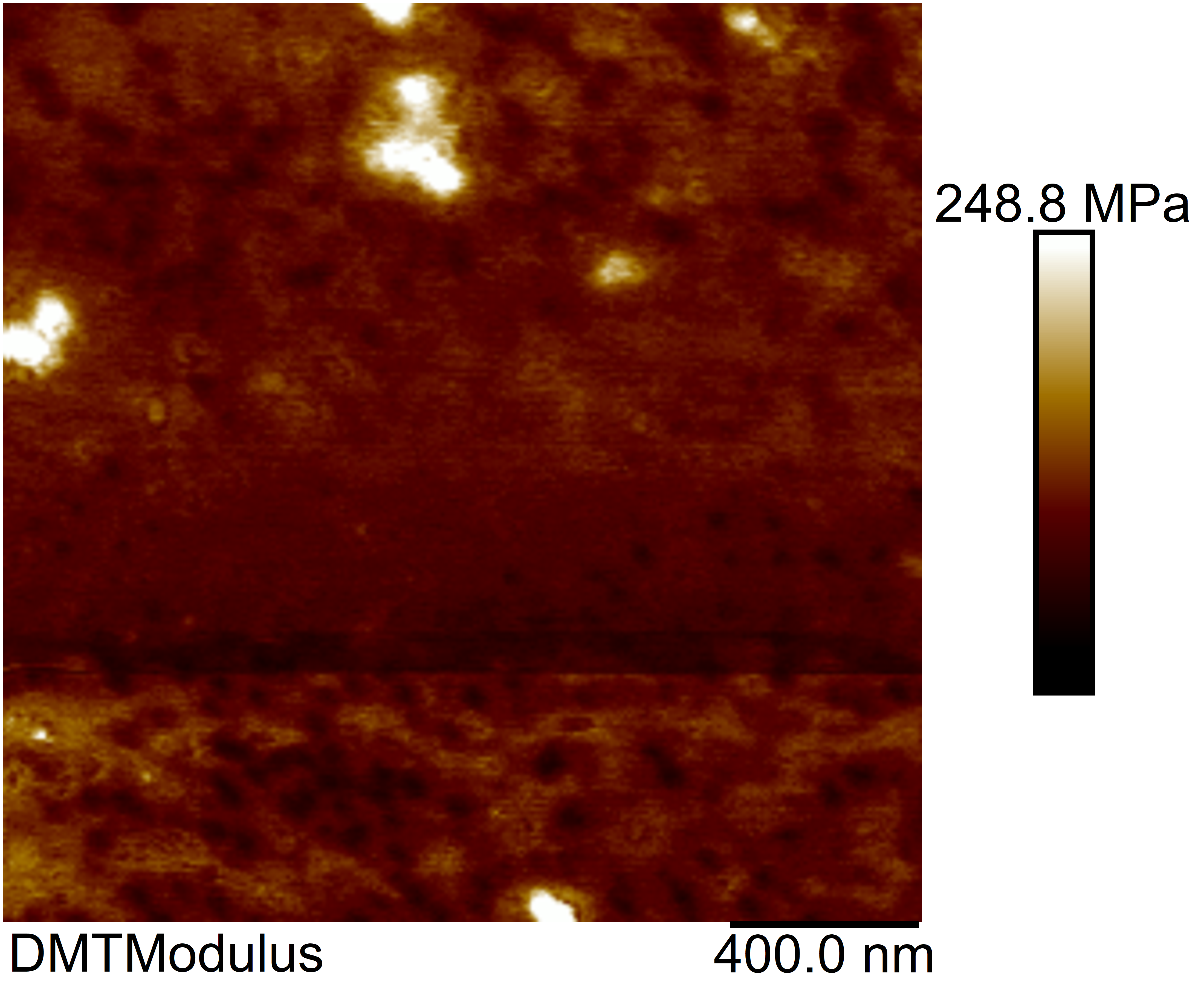


**Fig. S14** Young’s modulus mappings obtained by typical AFM imaging of PPM-PE films

The elastic modulus of PPM-PE film was measured by peakforce quantitative nanomechanics (QNM) mode of dimension icon atomic force microscope (AFM) and fitted by Derjaguin-Muller-Toporov (DMT) mode. As shown in **Fig. S14**, PPM-PE film delivers a high Young’s modulus (147 MPa), which is favorable for suppressing excessive volume expansion of SiO_x_ electrodes.


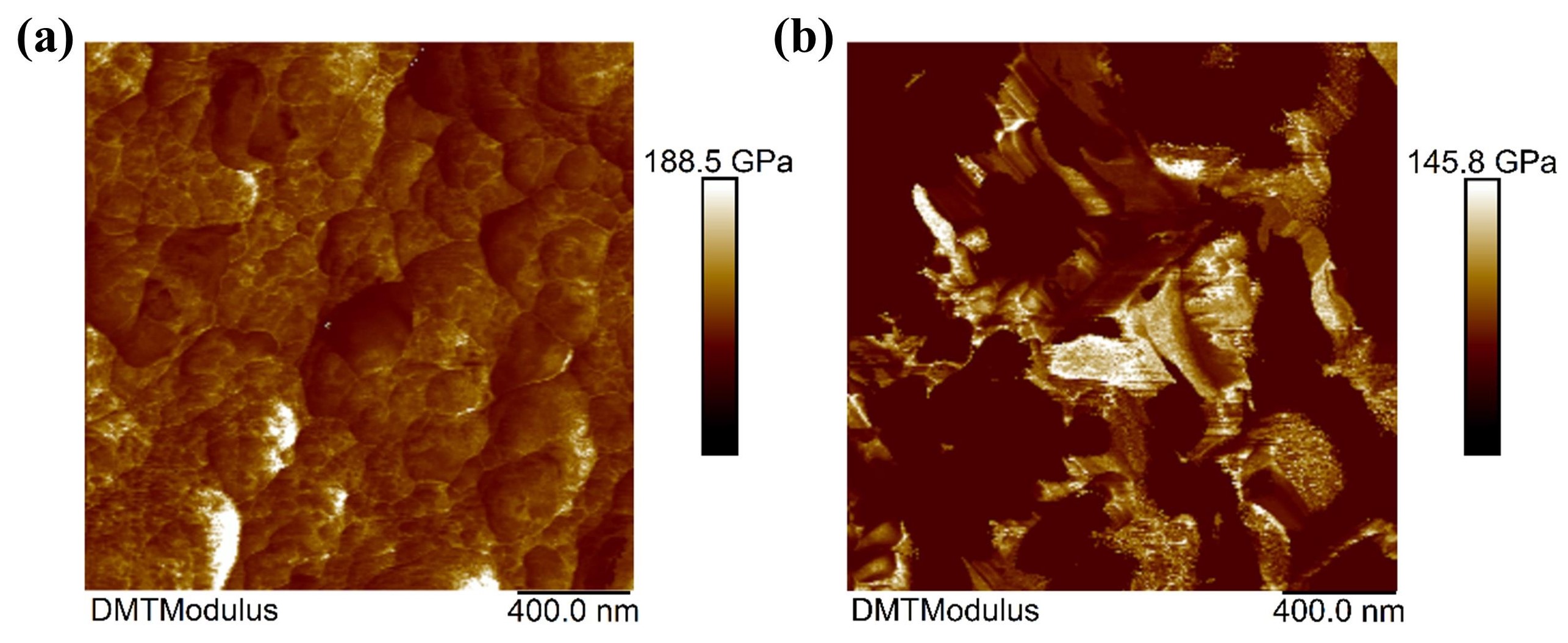


**Fig. S15** AFM DMT modulus mappings of a) SiO_x_@PPM-PE, b) SiO_x_@LE after 100 cycles at 0.5 C rate


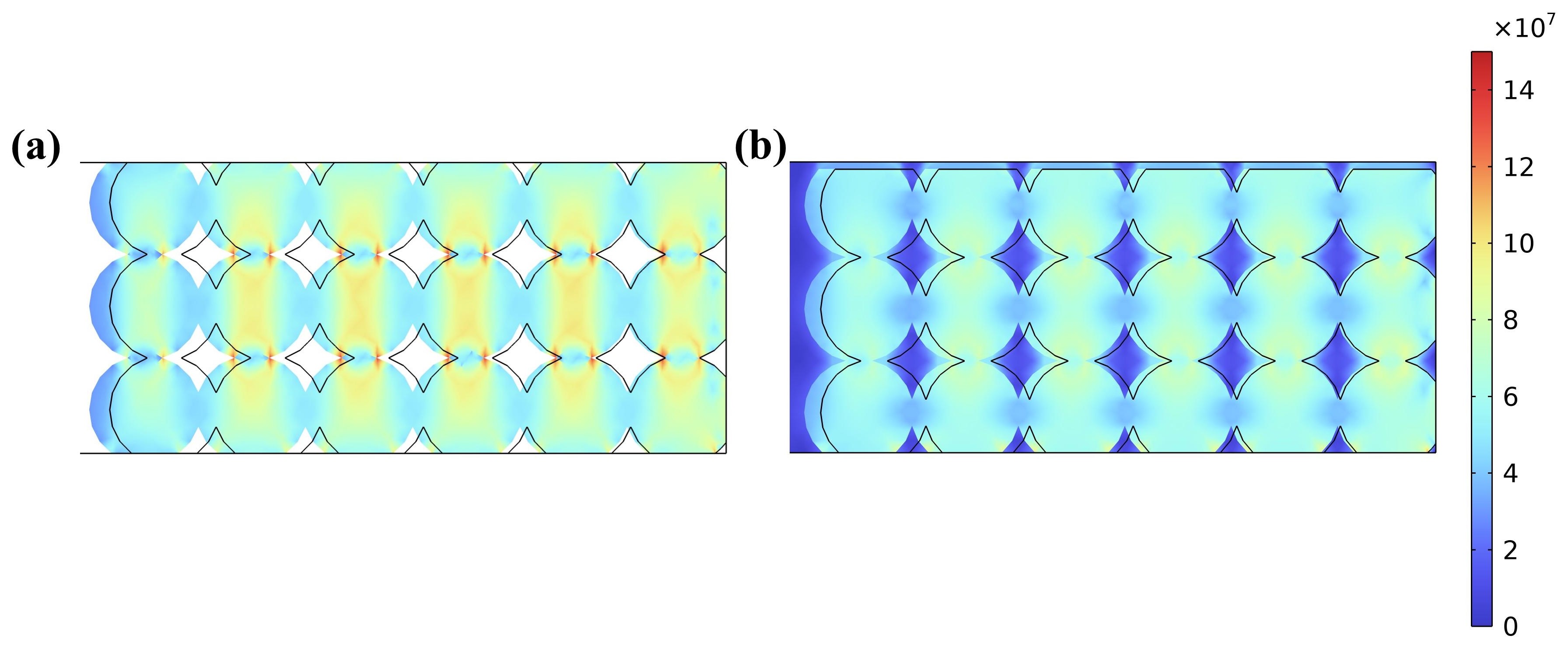


**Fig. S16** Enlarged stress distribution of SiO_x_ electrodes in the presence of (**a**) LE and (**b**) PPM-PE

Finite element simulation was used to investigate the effect of the mechanical properties of the electrolyte on the stress distribution of SiO_x_ electrodes. **Figure S16** shows that the stress on the surface of SiO_x_ electrodes with PPM-PE wrapped around is much lower than that using LE.


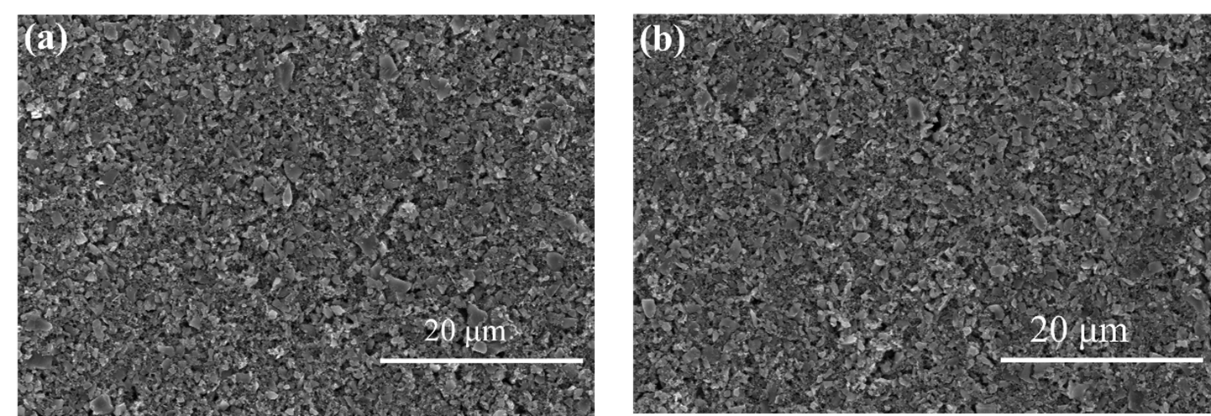


**Fig. S17** Top-viewed SEM images of **a**) SiO_x_@PPM-PE, **b**) SiO_x_@LE before cycling

As shown in **Fig. S17a-b**, the pristine SiO_x_ electrode surface using LE or PPM-PE shows no apparent cracks.


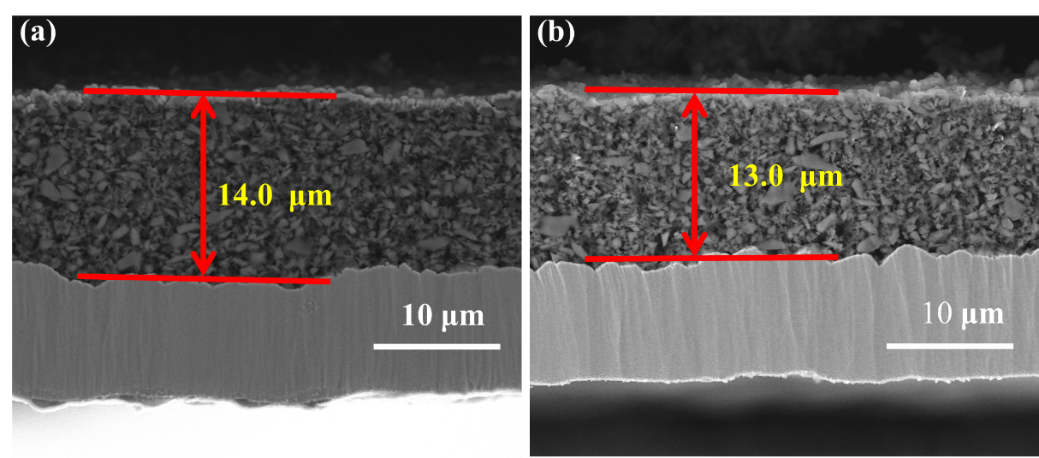


**Fig. S18** Cross-sectional SEM images of **a**) SiO_x_@PPM-PE, **b**) SiO_x_@LE before cycling


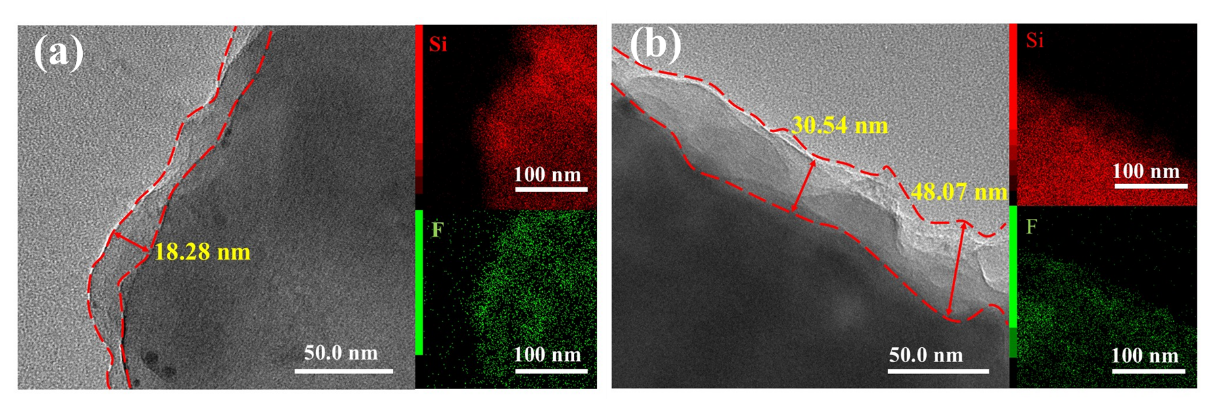


**Fig. S19** TEM imaging and corresponding elemental mapping images of SiO_x_ particles from **a**) SiO_x_@PPM-PE, **b**) SiO_x_@LE after 50 cycles at 0.5 C rate

As shown in **Fig. S19**, TEM imaging and corresponding elemental mapping images of SiO_x_ electrodes with different electrolytes after 50 cycles at 0.5 C rate reflect the thickness and element composition of SEI layers on the surface of SiO_x_ particles after cycling.


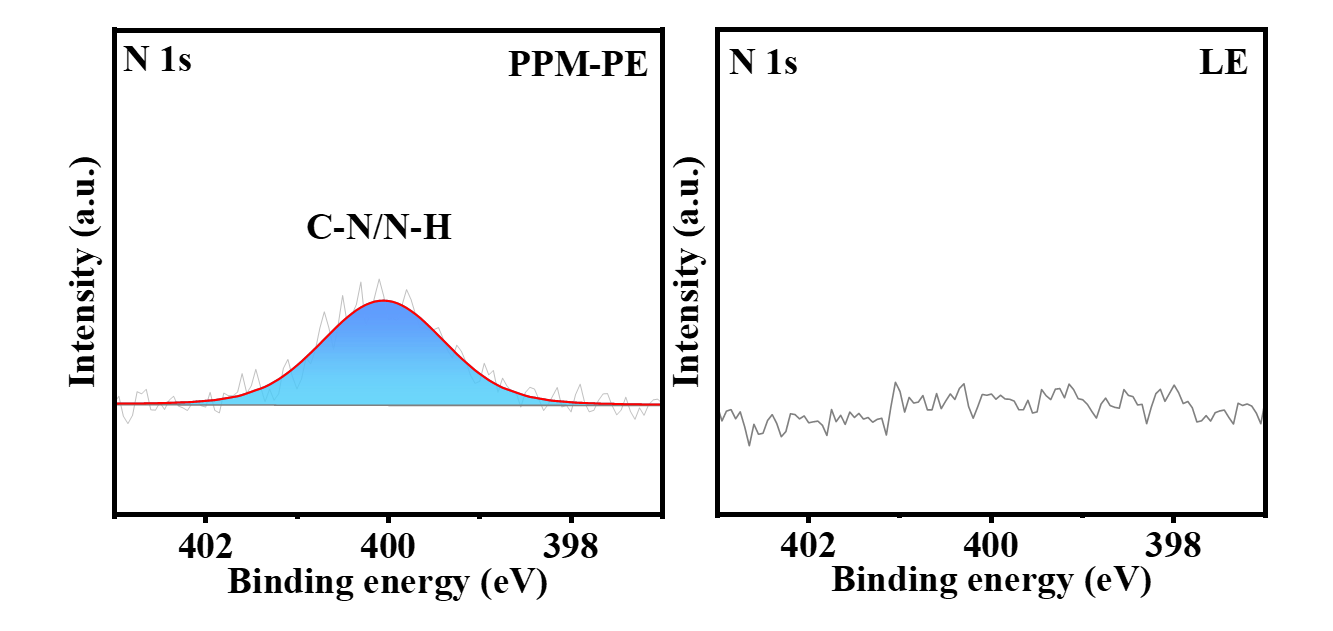


**Fig. S20** XPS spectra in N 1s branches of SiO_x_ electrodes after 30 cycles at 0.5 C rate

There is C–N/N–H type species found on the surface of SiO_x_ electrodes cycled with PPM-PE, indicating that PPM-PE may participate in the formation of SEI layers.


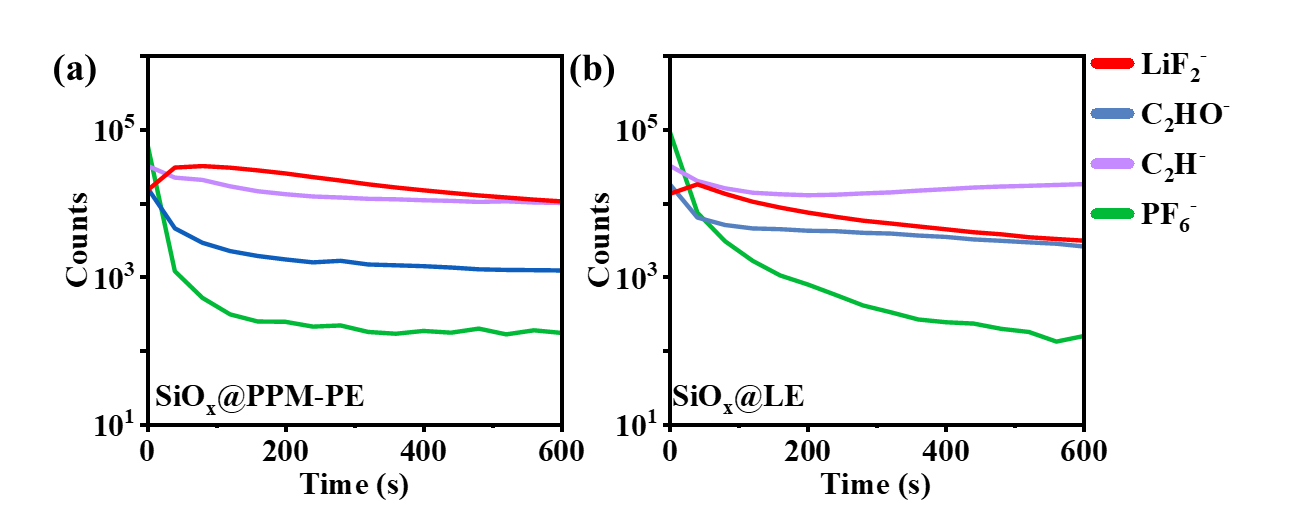


**Fig. S21** TOF-SIMS depth profiles of **a**) SiO_x_@PPM-PE, **b**) SiO_x_@LE

More LiF_2_^–^ and less C_2_HO^−^/C_2_H^–^ contents can be found on the SiO_x_@PPM-PE surface, compared with SiO_x_@LE.


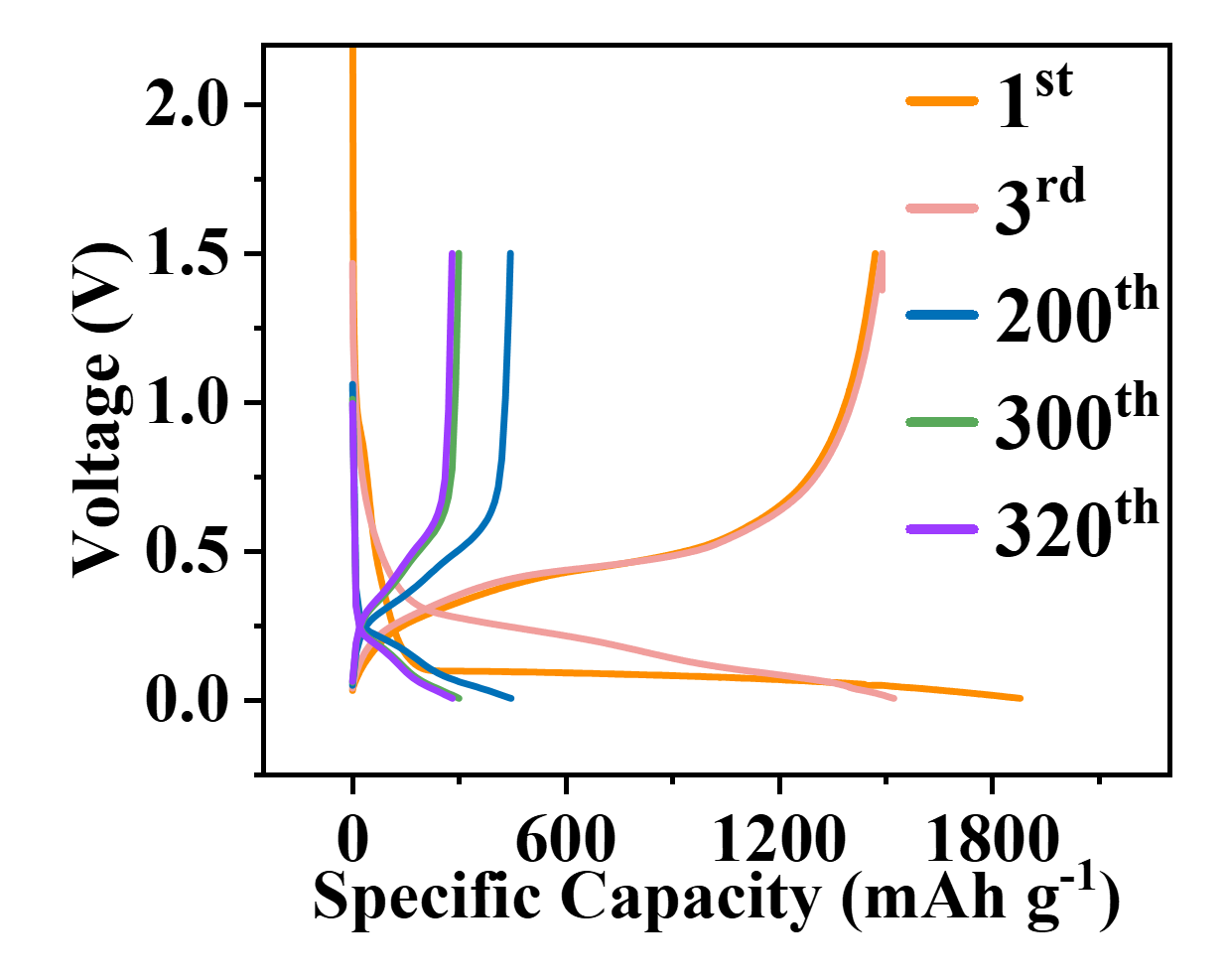


**Fig. S22** Charge-discharge curves of the SiO_x_/LE/Li half-cells at 0.5 C rate after 2 cycles of activation at 0.1 C rate, for the 1^st^, 3^rd^, 200^th^, 300^th^ and 320^th^ cycles of the cycle


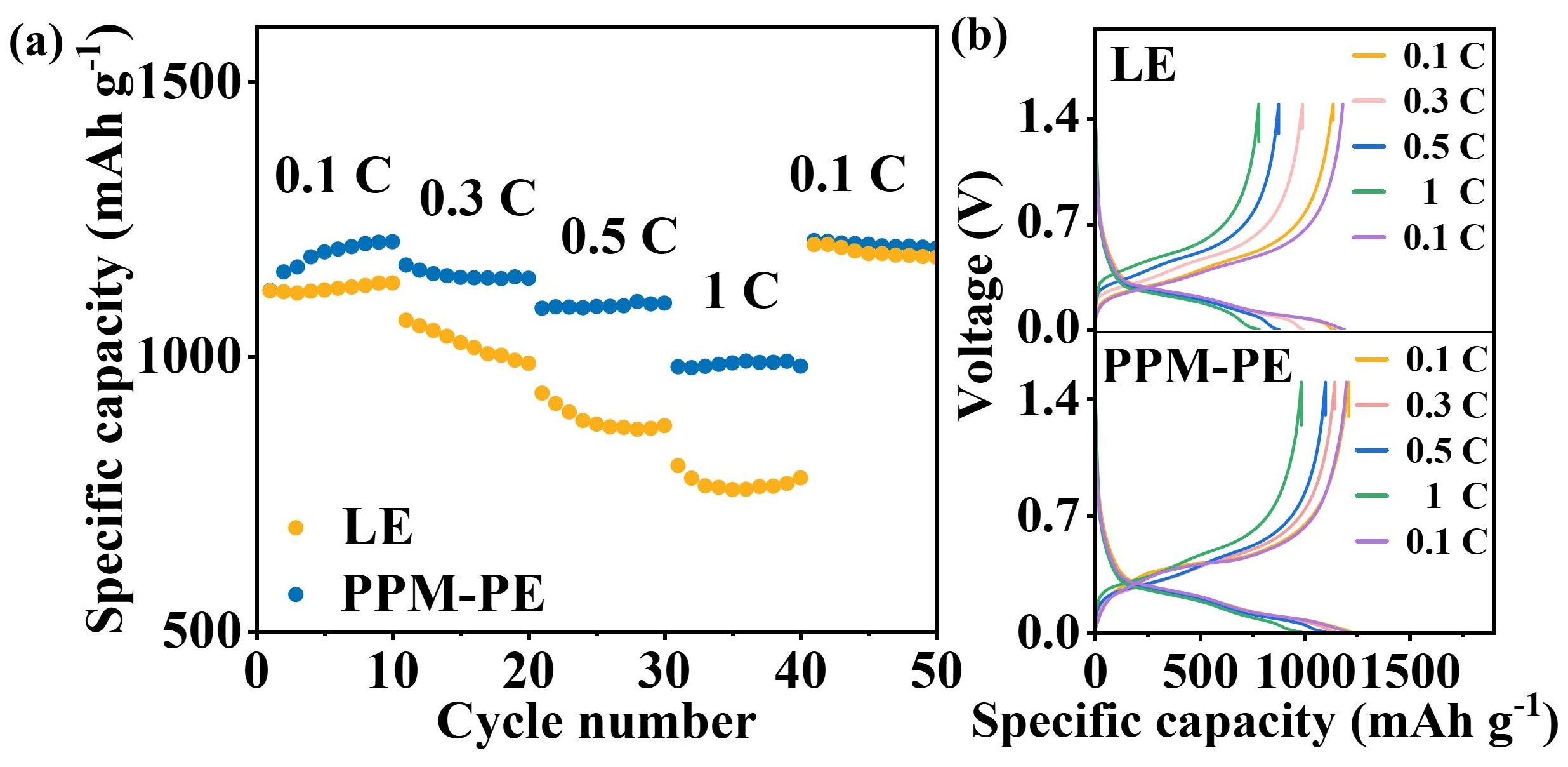


**Fig. S23 a)** Rate performance of different electrolytes in SiO_x_ electrode-based half-cells and **b**) charge-discharge curves of SiO_x_ at different rates for the final cycle of the varied rate

As presented in **Fig. S23**, PPM-PE delivers higher delithiation capacities than LE at different current densities from 0.1–1 C, demonstrating faster electrochemical reaction kinetics of SiO_x_ electrodes when using PPM-PE.


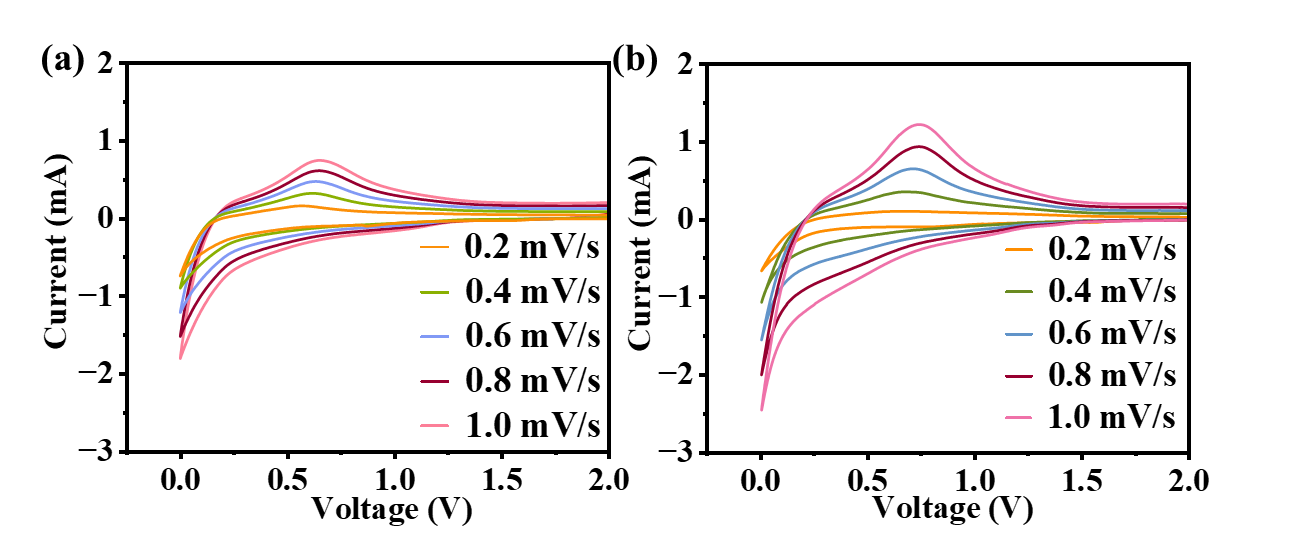


**Fig. S24** CV curves of **a**) LE, **b**) PPM-PE at different scan rates

**Fig. S24** compares the cyclic voltammetry (CV) curves of different electrolytes at varied scan rates from 0.2 to 1.0 mV s^‒1^.


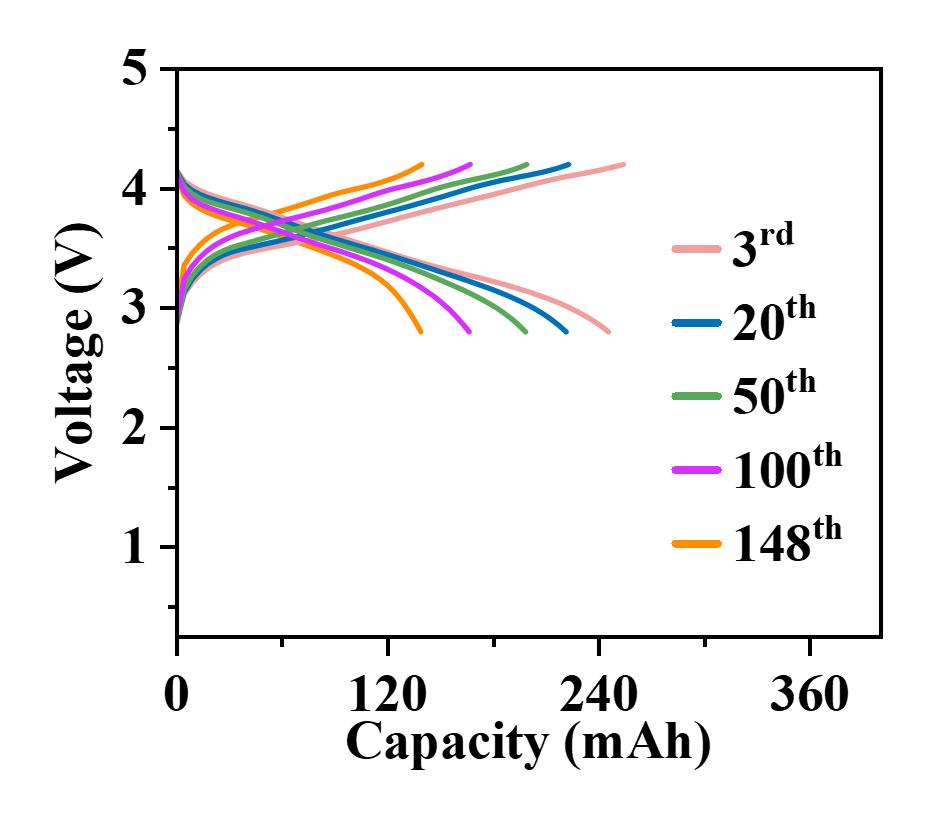


**Fig. S25** Charge and discharge curves of the NCM811/LE/SiO_x_ soft package full cell at 1 C rate for varied cycles


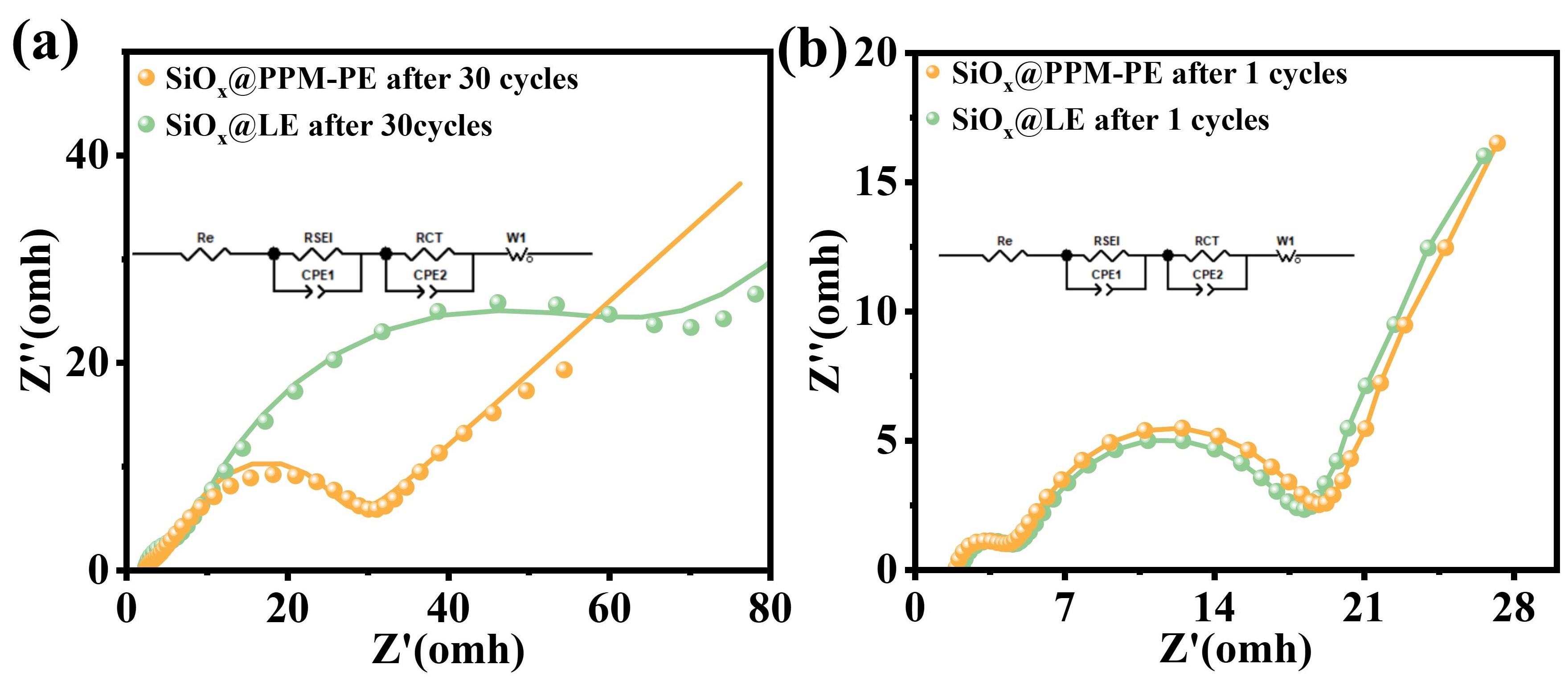


**Fig. S26** Nyquist curves of SiO_x_ electrodes in half-cells with different electrolytes after **a**) the initial cycle and **b**) 30 cycling. (Inset is an equivalent circuit fitted by Zview software). The dots and lines represent experimental data and fitted data, respectively.

As shown in **Fig. S26**, each EIS curve consists of two semicircles corresponding to the interfacial resistance and charge transfer resistance (denoted as R_SEI_ and R_CT_, respectively) at the electrode/electrolyte interface, and shows a straight line at the low frequency region corresponding to Li^+^ diffusion in the active particles. The Zview software was used to construct an equivalent circuit diagram to fit the impedance spectrum, which can give the values of R_SEI_ and R_CT_ (Tables S2 and S3). The EIS results demonstrate that after the first cycle, all the half-cells show little difference R_SEI_ and R_CT_. After 30 cycles, the R_SEI_ and R_CT_ increase (ΔR_SEI_ and ΔR_CT_, respectively) of half-cells with SiO_x_ @PPM-PE are only 0.1 and 12.2 Ω, respectively. In a fair comparison, the half-cell with SiO_x_ @LE exhibits more significant increases, with R_SEI_ and R_CT_ reaching 1.9 and 53.3 Ω, respectively. This finding indicates improved stability of SEI in half-cells when using SiO_x_ @PPM-PE during cycling.


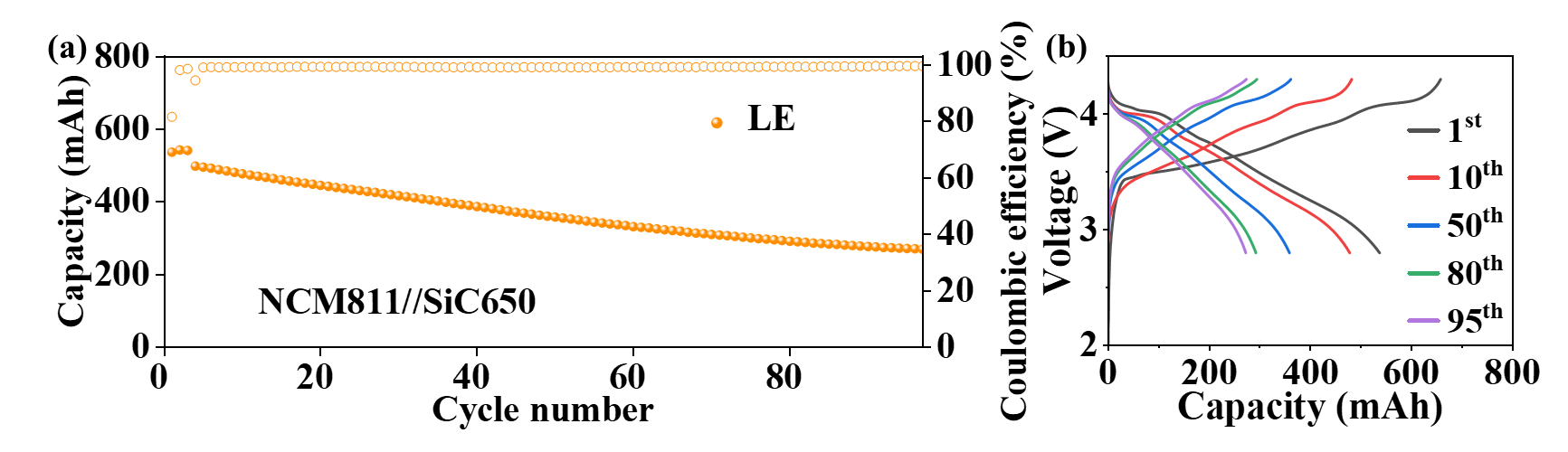


**Fig. S27** **a**) Cycling performance of NCM811/LE/SiC650 soft package full cells at 0.4 C rate between 2.8 to 4.3 V and the corresponding **b**) charge-discharge curves at varied cycles

As exhibited in **Fig. S27**, the assembled cell provides a capacity retention of 54.62% and an average Coulombic efficiency of 99.00% after 95 cycles at 0.4 C rate between 2.8 and 4.3 V.

**Table S1** Cycle performance comparison of SiO_x_ electrode based half-cells using PPM-PE and previously reported typical electrolytes

|  | Electrolytes | Remaining specific capacity (mAh g^−1^) | Cycle  number | Capacity  Retention (%) | References |
| --- | --- | --- | --- | --- | --- |
| Li//SiO_x_  half cells | PPM-PE | 1035 | 400 cycles at 0.5C | 80.63 | Our work |
|  | 2FPI-5FEC | 895.3 | 100 cycles | 69.20 | [S1] |
|  | 10.0 TFE | 1135 | 100 cycles | 69.90 | [S2] |
|  | LTN | 1013 | 100 cycles | 83.20 | [S3] |
|  | TMVS | 1017 | 200 cycles | 70.00 | [S4] |
|  | SE + 0.5 F | 1250 | 50 cycles | 82.40 | [S5] |
|  | 15.0 TTFP | 1302 | 100 cycles | 84.90 | [S6] |
|  | VL-2 | 1179 | 100 cycles | 72.70 | [S7] |

**Table S2** *R*_SEI_ and *R*_CT_ of SiO_x_ electrodes in half-cells with different electrolytes after initial cycling

|  | *R*_SEI_ (Ω) | *R*_CT_ (Ω) |
| --- | --- | --- |
| SiO_x_@PPM-PE | 4.5 | 19.0 |
| SiO_x_@ LE | 4.4 | 18.2 |

**Table S3** *R*_SEI_ and *R*_CT_ of SiO_x_ electrodes in half-cells with different electrolytes after 30th cycling

|  | *R*_SEI_ (Ω) | *R*_CT_ (Ω) |
| --- | --- | --- |
| SiO_x_@PPM-PE | 4.6 | 31.2 |
| SiO_x_@ LE | 6.3 | 71.5 |

**Supplementary References**

1. R. Li, B. Cui, Q. Zhou, X. Mu, Y. Gao et al., Stabilizing electrode-electrolyte interface for high-performance SiO anode by dual electrolyte additive. J. Energy Chem. **86**, 32-40 (2023). <https://doi.org/10.1016/j.jechem.2023.07.015>
2. H.N. Kim, T. Yim, 1,1,2,2-tetrafluoroethyl-2,2,3,3-tetrafluoropropyl ether as an advanced electrolyte additive for SiO_x_-based lithium-ion batteries. J. Alloys Compd. **931**, 167529 (2023). <https://doi.org/10.1016/j.jallcom.2022.167529>
3. Y.-F. Tian, S.-J. Tan, Z.-Y. Lu, D.-X. Xu, H.-X. Chen et al., Insights into anion-solvent interactions to boost stable operation of ether-based electrolytes in pure-SiO_x_||LiNi_0.8_Mn_0.1_Co_0.1_O_2_ full cells. Chem. Int. Ed. **62**(33), e202305988 (2023). <https://doi.org/10.1002/anie.202305988>
4. C.C. Nguyen, H. Choi, S.-W. Song, Roles of oxygen and interfacial stabilization in enhancing the cycling ability of silicon oxide anodes for rechargeable lithium batteries. J. Electrochem. Soc. **160**(6), A906-A914 (2013). <https://doi.org/10.1149/2.118306jes>
5. J.W. Kim, M.J. Seong, D.W. Park, G. Jeong, T. Yim, Anti-corrosive and surface-stabilizing functional electrolyte containing LiFSI and LiPO_2_F_2_ for SiO /NCM811-based batteries. Corros. Sci. **198,** 110117 (2022). <https://doi.org/10.1016/j.corsci.2022.110117>
6. S. Lee, T. Yim, Regulating interfacial stability of SiO_x_ anode with fluoride-abundant solid–electrolyte interphase by fluorine-functionalized additive. Rare Met. **43**(2), 671-681 (2023). <https://doi.org/10.1007/s12598-023-02474-y>
7. H.N. Kim, S.Y. Kim, J. Ahn, T. Yim, Simultaneous realization of multilayer interphases on a ni-rich NCM cathode and a SiO_x_ anode by the combination of vinylene carbonate with lithium difluoro(oxalato)borate. ACS Appl. Mater. **16**(12), 14940-14953 (2024). <https://doi.org/10.1021/acsami.4c01032>
